# Numerical study on the hydrodynamics of thunniform bio-inspired swimming under self-propulsion

**DOI:** 10.1371/journal.pone.0174740

**Published:** 2017-03-31

**Authors:** Ningyu Li, Huanxing Liu, Yumin Su

**Affiliations:** 1 Science and Technology on Underwater Vehicle Laboratory, Harbin Engineering University, Harbin, China; 2 Beijing Institute of Specialized Machinery, Beijing, China; University at Buffalo - The State University of New York, UNITED STATES

## Abstract

Numerical simulations are employed to study the hydrodynamics of self-propelled thunniform swimming. The swimmer is modeled as a tuna-like flexible body undulating with kinematics of thunniform type. The wake evolution follows the vortex structures arranged nearly vertical to the forward direction, vortex dipole formation resulting in the propulsion motion, and finally a reverse Kármán vortex street. We also carry out a systematic parametric study of various aspects of the fluid dynamics behind the freely swimming behavior, including the swimming speed, hydrodynamic forces, power requirement and wake vortices. The present results show that the fin thrust as well as swimming velocity is an increasing function of both tail undulating amplitude *A*_*p*_ and oscillating amplitude of the caudal fin *θ*_*m*_. Whereas change on the propulsive performance with *A*_*p*_ is associated with the strength of wake vortices and the area of suction region on the fin, the swimming performance improves with *θ*_*m*_ due to the favorable tilting of the fin that make the pressure difference force more oriented toward the thrust direction. Moreover, the energy loss in the transverse direction and the power requirement increase with *A*_*p*_ but decrease with *θ*_*m*_, and this indicates that for achieving a desired swimming speed increasing *θ*_*m*_ seems more efficiently than increasing *A*_*p*_. Furthermore, we have compared the current simulations with the published experimental studies on undulatory swimming. Comparisons show that our work tackles the flow regime of natural thunniform swimmers and follows the principal scaling law of undulatory locomotion reported. Finally, this study enables a detailed quantitative analysis, which is difficult to obtain by experiments, of the force production of the thunniform mode as well as its connection to the self-propelled swimming kinematics and vortex wake structure. The current findings help provide insights into the swimming performance and mechanisms of self-propelled thunniform locomotion.

## 1 Introduction

Despite impressive innovations in underwater vehicles, both the military and scientific communities are expecting to benefit from vehicles with better performance, and the biomimetic propulsion system which applies principles abstracted from fish swimming has been increasingly used [1–4]. Fish which primarily employ body and/or caudal fin (BCF) swimming mode for propulsion and maneuvering are divided into five families in accordance with the manner they swim: anguilliform, sub-carangiform, carangiform, thunniform and ostraciiform [5–7]. For thunniform fish, the undulation is limited to the rear 1/3 of the body and reaches the maximal amplitude at the end of the tail peduncle [8, 9]. As the main propulsive device, driven by the tail peduncle, the caudal fin sways and oscillates [10, 11]. As a primary locomotion mode for many fast moving swimmers, thunniform swimming has attracted increasing attention.

A multitude of experiments have provided a wealth of data in terms of wake structure and body kinematics for undulatory swimming. A single row wake structure has been found for carangiform and thunniform swimmers (*e*.*g*. Triantafyllou and Triantafyllou [12], Muller *et al*. [13] and Nauen and Lauder [14]), while a double row wake structure has been found in the wake of anguilliform fishes (*e*.*g*. Muller *et al*. [15], Tytell and Lauder [16] and Hultmark *et al*. [17]). Fish and Lauder [18] have indicated that the wake structure is different between eel-like (anguilliform) fishes and fishes with a narrow tail peduncle region (*e*.*g*. thunniform fishes). In our previous experiment [19], larger oscillation period and amplitude were observed for the cyprinid as compared to the bulltrout. However, experimental studies are limited in their ability of experimental control over fish swimming, as shown by Tytell [20]. Another relevant problem arises from the difficulties in obtianing locomotor forces and the swimming efficiency based on the wake measured [21]. These factors indicate the challenges for experiments alone to give conclusive insights into the hydrodynamics of BCF locomotion.

Fortunately, numerical simulations that are carefully designed for controlled numerical studies can be used for complementing live-fish experiments. Wolfgang *et al*. [22] simulated the flow past a giant danio, and numerical results matched well with their experiment. The research of Zhu *et al*. [10] on swimming tuna and giant danio has shown two vorticity interaction modes: the constructive mode and the destructive mode. Borazjani and Sotiropoulos [23, 24] investigated the hydrodynamics of carangiform and anguilliform modes in the transitional and inertial flow regimes, and analyzed the swimming performance and wake structure of the two motion patterns. The virtual thunniform swimmer with three types of caudal fin models was investigated in [25], and simulations showed the crescent-shaped fin was the most efficient. In addition to the aforementioned, many existing numerical studies on the hydrodynamics of fish swimming are also conduceted under a non-self-propelled condition (*e*.*g*. Liu *et al*. [26, 27], Yang and Su [2] and Lee *et al*. [28]), where the fish is immersed in a uniform incoming flow or swimming at a constant velocity. However when a fish swims autonomously, dynamical behaviors are purely determined by hydrodynamic forces and the fluid-fish interaction [29]. This is also the case for flying animals as Ben-Gida *et al*. [30] have presented. Wu [31] has indicated that the fluid dynamic performance of self-propelled locomotion is different from that of steady forward swimming.

It is therefore necessary to carry out self-propelled numerical simulations to better understand mechanisms of BCF swimming mode. At present, many studies in this area [32–43] are concentrated on self-propelled oscillating foils/fins. These investigations have provided some insights into the mechanisms of oscillating-based locomotion by fish fins, but are limited by the absence of the adjacent body. Thus, computations of flow past three-dimensional (3D) flexible fishes in BCF mode are desirable. Carling *et al*. [44], Kern and Koumoutsakos [45], Hu *et al*. [46], Katumata *et al*. [47] and van Rees *et al*. [48] simulated self-propelled anguilliform swimming. In their investigations, the swimming speed was not prescribed beforehand but obtained as a solution, and a quantitative study of the body motion, wake flow field and hydrodynamic forces was provided. Borazjani and Sotiropoulos [29] found that anguilliform kinematics not only achieves faster speed but also has higher efficiency in the viscous and transitional regimes, and however carangiform body reaches larger velocity in all regimes but is more efficient in the inertial regime only. Yang *et al*. [49] presented an integrated numerical approach to examine a self-propelled sub-carangiform fish. Borazjani and Daghooghi [50] observed that the formation of an attached vortex at the leading edge of the caudal fin for a mackerel body (carangiform mode) with various tail shapes. Xia *et al*. [51] investigated the hydrodynamics under self-propulsion using a tuna-inspired computational model with thunniform kinematics.

These above numerical investigations have produced significant results and provided understanding/knowledge in the hydrodynamics of several aquatic motion patterns. However currently, numerical investigations on 3D multi-degree of freedom (DoF) thunniform swimming under self-propulsion are scarce. Additionally, systematic parametric studies of the swimming speed, hydrodynamic forces, propulsive efficiency and wake structure of thunniform mode have not been well addressed in the literature, since keeping one of parameters constant while changing others in a repeatable manner is hard to achieve in live-fish experiments [8, 52–54]. Moreover, detailed quantitative numerical studies are desired to be conducted in the context of the flow regime and scaling law of natural thunniform swimmers to complement related experiments and reveal self-propelled mechanisms.

In this paper, we extend our previous studies on tuna-like thunniform swimming at the steady state [2] or with 1-DoF in the forward direction [55], to 3-DoF self-propelled thunniform simulations. Our attention is firstly focused on the computational model set-up of the 3D flexible fish with an undulating body and an oscillating caudal fin. Then based on a fluid-structure interaction (FSI) method, the swimming performance and mechanisms during the process where the fish accelerates from static state to steady cruise are analyzed in detail. Following this, we have carried out a systematic parametric study of the hydrodynamics behind the self-propelled behavior. This allows us to address the practical question of how the propulsive properties of the thunniform swimming mode are expected to change with variations in key governing parameters. Finally, based on the present numerical simulations, we comment on the overall hydrodynamics of the self-propelled thunniform swimming.

## 2 Problem description and numerical method

### 2.1 Computational model

**Fig 1:**
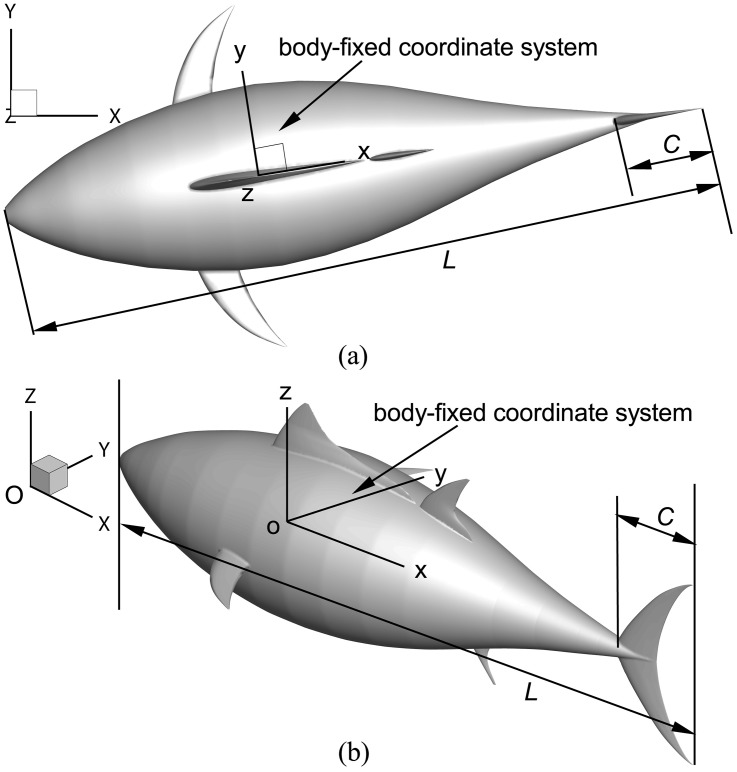
Physical model and coordinate systems. (a) Top view and (b) perspective view.

In the current study, we use the fish geometry representing a tuna to investigate the hydrodynamics of 3D flexible body performing thunniform bio-inspired swimming. We use the form of the “*FangSheng-I*”, a laboratory robotic fish mimicking the geometry and motion of a tuna [2, 56]. The total length of the fish (from the head to the caudal fin) *L* is 2.4 m and the maximal width of the main body (without the fins) is 0.62 m. The swimmer has a tuna-like lunate caudal fin with cross-sections of NACA 0018 shape. The chord length of the caudal fin *C*, defined as the distance from the leading edge point to the line connecting two tail tips, is 0.34 m. Fig 1 shows the physical model of the fish and the coordinate systems. We employ an global coordinate system *O*-*XYZ* fixed in space and a body-fixed coordinate system *o-xyz*, where the *x*-axis points to the fish tail along the stretched-straight mean line, *y-*axis the right side along the transverse direction, and *z-*axis the upper part of fish body.

In general, thunniform swimmers are on the higher end of marine food chain and living by hunting other fish species, such as tunas and billfishes, etc., and thus in most cases they have a strong and big front body [57] of high rigidity. When cruising, the front 2/3 of their body has little undulation and deformation, as shown by Dewar and Graham’s research [8] and the observation of Fish *et al*. [9]. Although the fish body and tail are usually treated together as a single undulating object, *i*.*e*., the body traveling wave passes through the caudal fin [23, 58], it is interesting to note that in thunniform fishes the caudal fin may undergo kinematic behaviors independent of the body [18, 53, 59, 60]. Therefore, herein the prescribed kinematics of the thunniform swimmer consists of two basic components: one is the undulation of the rear 1/3 of the body and the other is the motion of the caudal fin.

#### 2.1.1 Undulation of flexible rear body

We choose the kinematics for the thunniform body undulation as employed in the experiment of robotic tuna by Barrett *et al*. [61], which closely emulates that found by swimming observations of Dewar and Graham [8] on real tunas. The transverse undulation of the rear 1/3 of fish body is described by the following equation:

y(x,t)=A(x)sin(2πft−kx), (1)

where *f* is the motion frequency, *t* is the time, *k* is the wave number and *A*(*x*) is the amplitude envelope, usually approximated by a quadratic function (*e*.*g*. the experiment of Barrett *et al*. [61] and the simulation of Xia *et al*. [51] by virtual thunniform fish). In our study, the amplitude envelope is given the form of a polynomial with adjustable order,

$$A(x)=A_{p}{\left(\frac{x-x_{r}}{l_{r}}\right)}^{\alpha },$$ (2)

where *x*_*r*_ denotes the *x* coordinate at the beginning of the rear 1/3 of the body, where the undulating amplitude is minimum; *l*_*r*_ denotes the length of the rear 1/3 of the body; *A*_*p*_ is the undulating amplitude of the tail peduncle end at which the amplitude is maximum; *α* is the order of the shape function, adjustable to attain a specific form of *A*(*x*).

In the present work *α* is taken as 2, which coincides with previous studies on typical thunniform swimmers [51, 61]. In order to match with previous numerical and experimental work in our laboratory [2, 56], the wave number *k* is set at zero in the current simulations. The effect of *k* on the hydrodynamics will be discussed in our future research.

However, if we simply employ Eq (1) to describe the undulation of the flexible rear body, the fish body would be elongated during movement, just as the results shown in a number of past numerical studies [2, 10, 22, 51] on thunniform bio-inspired swimming. In view of this, non-elongating swimmers are considered in the studies of Tytell *et al*. [62] and Gazzola *et al*. [63] on anguilliform locomotion. Actual fish have a multitude of vertebrae which function as many joints to smoothly generate an undulating motion while they are swimming [18, 64]. Inspired by this, in the current study, we harness enough links to closely emulate the undulation of the rear 1/3 of the body in thunniform fishes, and ensure the unchanging length of the swimmer by keeping the length of every link unchanged during deformation.

**Fig 2:**
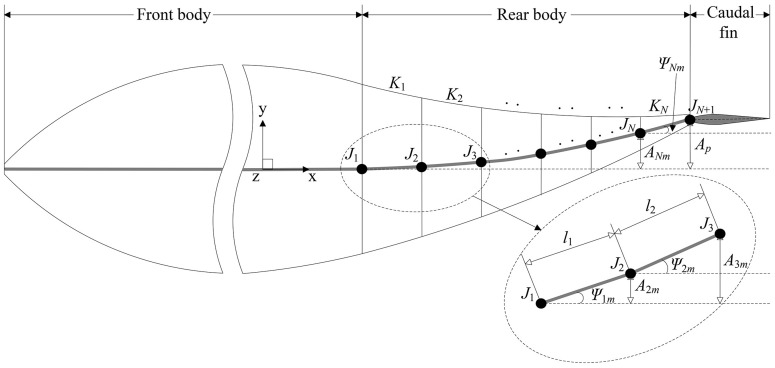
Diagram of body undulation. Details of the area around the first two links are given by a closer look.

The flexible rear body is segmented into *N* discrete links, all expressed by *K*_*i*_ (*i* = 1, 2, …, *N*), as shown in Fig 2. Links are connected by joints expressed by *J*_*i*_ (*i* = 1, 2, …, *N+*1). The motion of each link can be described as the sum of translational motion along with and the rotational motion around (the rotation axis is parallel to the *z-*axis) the end of last link. Let the length of link *K*_*i*_ be *l*_*i*_ (not time-varying), and the coordinates of joint *J*_*i*_ in the *xoy* plane be (*J*_*ix*_ (*t*), *J*_*iy*_ (*t*)). The translational motion and rotational motion of link *K*_*i*_ can be expressed by

$$\{\begin{array}{c} y_{i}(t)=A_{im}\text{sin}(2\pi ft)\\ \psi_{i}(t)=\psi_{im}\text{sin}(2\pi ft) \end{array},\;i=1,\;2,\;\ldots ,\;N,$$ (3)

where *A*_*im*_ and *ψ*_*im*_ are translational and rotational amplitudes of link *K*_*i*_, respectively.

Considering the kinematics of a linked body, finally the translational amplitude, rotational amplitude and rotation center of each link in Eq (3) is given by

$$\{\begin{array}{l} \text{translational amplitude}:A_{1m}=0,\;A_{im}=A_{p}{\left(\frac{\sum_{q=1}^{i-1}l_{q}}{l_{r}}\right)}^{\alpha },\;i=2,\;\ldots ,\;N\\ \text{rotational amplitude}:\psi_{im}=\text{arcsin}\left(\frac{A_{(i+1)m}-A_{im}}{l_{i}}\right),\;i=1,\;2,\;\ldots ,\;N-1,\;\psi_{Nm}=\text{arcsin}\left(\frac{A_{p}-A_{Nm}}{l_{N}}\right)\\ \text{rotation center}:\left(\begin{array}{c} J_{1x}(t)\\ J_{1y}(t) \end{array}\right)=\left(\begin{array}{c} x_{r}\\ 0 \end{array}\right),\;\left(\begin{array}{c} J_{ix}(t)\\ J_{iy}(t) \end{array}\right)=\left(\begin{array}{c} J_{(i-1)x}(t)+l_{i-1}\text{cos}\psi_{i-1}(t)\\ J_{(i-1)y}(t)+l_{i-1}\text{sin}\psi_{i-1}(t) \end{array}\right),\;i=2,\;\ldots ,\;N \end{array}.$$ (4)

**Fig 3:**
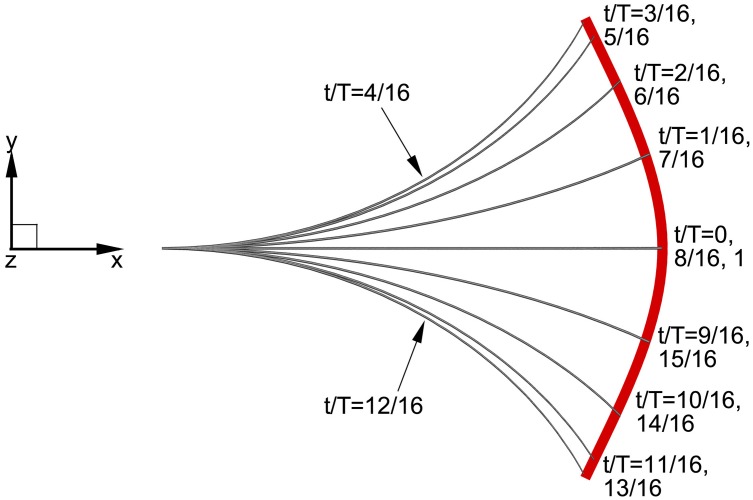
The midlines of the flexible, undulating body during one period. Since the front part of the swimmer makes no undulation, only the rear is shown. The red line shows the motion trajectory of the tail peduncle end.

The midlines of the thunniform fish determined by the above kinematic model for one motion cycle are presented in Fig 3, where the motion trajectory of the tail peduncle end constrained by unchanging body length is depicted by the red line and the swimmer’s wave-like motion in the rear 1/3 of its body has been well simulated.

#### 2.1.2 Kinematics of caudal fin

**Fig 4:**
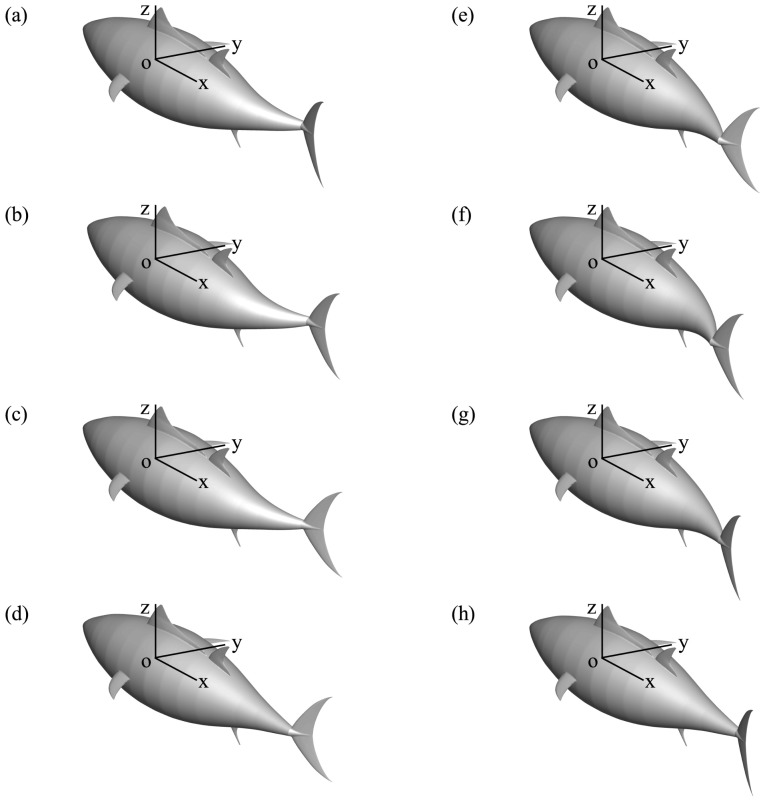
The prerscribed motion of the thunniform swimmer in one cycle. (a) *t*/*T* = 1/8, (b) 1/4, (c) 3/8, (d) 1/2, (e) 5/8, (f) 3/4, (g) 7/8 and (h) 1.0.

Based on the investigations of Barrett *et al*. [61] and Zhu *et al*. [10] on fish-like swimming and our previous studies [11, 65] on caudal fin hydrodynamics, the caudal fin perfroms a swaying motion which follows the flexible body undulation and a oscillating motion around the tail peduncle end. The kinematic description is in agreement with the observation of tuna swimming by Dewar and Graham [8] and Donley and Dickson [53]. Therefore, we have the equations for swaying and oscillating motions of the caudal fin as follows:

$$\{\begin{array}{l} y_{f}(t)=A_{p}\text{sin}(2\pi ft)\\ \theta (t)=\theta_{m}\text{sin}(2\pi ft-\varphi ) \end{array},$$ (5)

where the swaying amplitude of the caudal fin is equal to the undulating amplitude of the tail peduncle end *A*_*p*_ since the swaying motion of the caudal fin is driven by the tail peduncle. For this study the phase difference *φ* between swaying and oscillating motions is always 90° [2, 66] and *θ*_*m*_ is the oscillating amplitude of the caudal fin. Finally as a combination of the specified body and caudal fin motion, the postures of the fish for one swimming period *T* (defined as *T* = 1/*f*) are displayed in Fig 4, in which the whole fish undergoes a rhythmic motion with constant body length.

### 2.2 Simulation of 3-DoF self-propelled swimming

#### 2.2.1 Numerical approach for fluid-structure system

We focus on the 3-DoF self-propelled motion of a deforming body into an incompressible viscous fluid. The equations governing this flow are the 3D unsteady, incompressible Navier-Stokes equations in a body-fixed coordinate system that is translating with a linear velocity ***u***_*T*_ and rotating with angular velocity **Ω** relative to the global coordinate system [67]:

$$\{\begin{array}{l} \nabla \cdot u_{r}=0\\ \frac{\partial u}{\partial t}+\nabla \cdot \left(u_{r}u\right)+\Omega \times (u-u_{T})=-\frac{1}{\rho }\nabla p+\frac{\mu }{\rho }\nabla^{2}u \end{array},$$ (6)

where ***u*** is the absolute velocity (the velocity viewed from the global coordinate system), ***u***_*r*_ is the relative velocity (the velocity viewed from the body-fixed coordinate system), *p* is the pressure, *ρ* is the fluid density and *μ* is the dynamic viscosity. For viscous flow, the following no-slip boundary condition needs to be imposed to supplement the fluid Eq (6):

$$u=u_{B},$$ (7)

where ***u***_*B*_ is the resultant velocity of the fish body. Generally, the motion of a flexible structure can be decomposed as a rigid whole-body motion and a deformation motion:

$$u_{B}=u_{T}+\Omega \times r+u^{\prime },$$ (8)

where ***r*** is the position vector from the center of mass. First two terms on the right-hand side of the above equation together make up the rigid velocity of the whole-body, are unknown prior to the resolution. ***u***′ is the specified velocity of deforming motion presented in Section 2.1.1 and 2.1.2 in the body-fixed coordinate system. In the current 3-DoF self-propelled simulation, the whole-body motion is obtained using the Newton’s motion (momentum) equations:

$$\{\begin{array}{l} m\frac{du_{TX}}{dt}=F_{X}\\ m\frac{du_{TY}}{dt}=F_{Y}\\ d(I_{Z}\Omega_{Z})=\Omega_{Z}\frac{dI_{Z}}{dt}+I_{Z}\frac{d\Omega_{Z}}{dt}=M_{Z} \end{array},$$ (9)

where *m* is the mass of the swimmer, *u*_*TX*_ and *u*_*TY*_ are the components of the translational velocity in *X* and *Y* direction, respectively, *F*_*X*_ and *F*_*Y*_ are the components of the hydrodynamic force in *X* and *Y* direction, respectively, *I*_*Z*_ is the moment of inertia about the yawing axis through the center of mass, Ω_*Z*_ is the yawing angular velocity and *M*_*Z*_ is the yawing moment.

Self-propelled swimming involves a coupled interaction procedure of fish body dynamics and unsteady hydrodynamics. Currently there are two methods to solve FSI problems: monolithic method [68–70] and partitioned method [71–73]. Herein we employ the partitioned method with an under-relaxation scheme, in which the fluid Eq (6) and structure Eq (9) are separately solved at each time-step. The coupling effects are embodied through the kinematic coordination condition (Eq (7)) and the dynamic boundary condition on the right-hand side of Eq (9), and an under-relaxation approach is adopted to stabilize the fluid-solid coupling.

**Fig 5:**
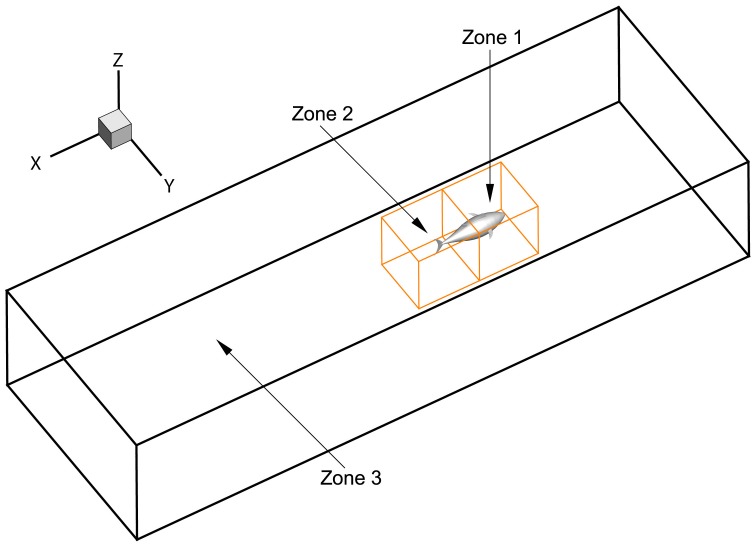
Computational domain. Zone 1 contains front body and Zone 2 contains rear body and caudal fin. Zone 3 denotes the large peripheral area that contains no fish.

**Fig 6:**
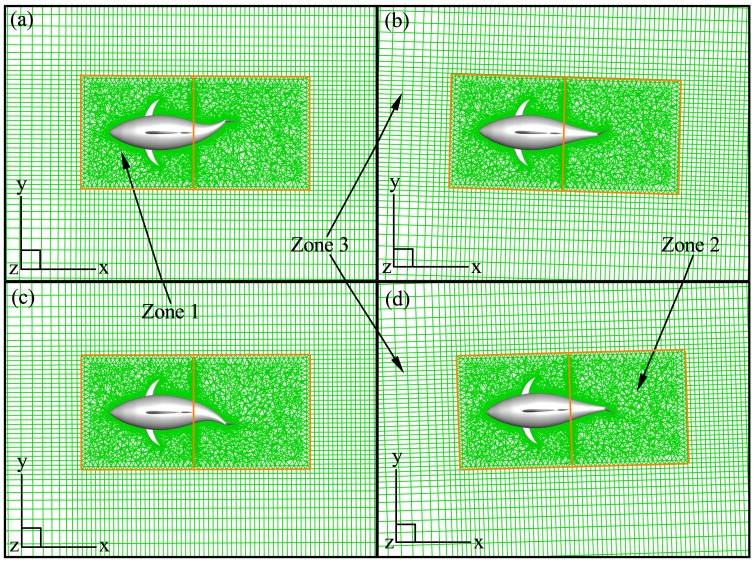
Mesh distribution for one swimming cycle. (a) *t*/*T* = 1/4, (b) 1/2, (c) 3/4, (d) 1.0.

Specifically, the fluid flow over the simmer is simulated by the commercial package FLUENT 16.1 with the pressure-based transient solver and second-order upwind spatial discretization. The second-order implicit discretization is employed for the time-stepping scheme and the no-slip wall boundary condition is used on the fish surface. An in-house DEFINE_GRID_MOTION macro is hooked to the main code of the FLUENT solver to achieve the desired deformation motion. The numerical computation of the Newton’s law equations governing rigid whole-body motion under the influence of hydrodynamic forces (moments) is carried out by an in-house user-defined function (UDF) in FLUENT. At each time step, the numerical procedure for the current fluid-structure system is the following:

1. Update the fish body position using the specified deformation velocity and the computed rigid whole-body velocity at time level *n* by Eq (8).
2. Advance the flow field past the swimmer to time level *n*+1 by solving the fluid Eq (6) with the no-slip boundary Condition (7) on the fluid-solid interface (the fish surface *S*).
3. Compute the fluid force and moment acting on the fish by the following under-relaxation scheme:
  $$\{\begin{array}{l} F_{X}^{n+1}=(1-\beta )F_{X}^{n}+\beta {\tilde{F}}_{X}^{n+1}\\ F_{Y}^{n+1}=(1-\beta )F_{Y}^{n}+\beta {\tilde{F}}_{Y}^{n+1}\\ M_{Z}^{n+1}=(1-\beta )M_{Z}^{n}+\beta {\tilde{M}}_{Z}^{n+1} \end{array},$$ (10)
  where *β* is the under-relaxation factor (0≤*β*≤1), which is chosen based on the trade-off between computational stability and efficiency, $\left(F_{X}^{n+1},F_{Y}^{n+1},M_{Z}^{n+1}\right)$ and $\left({\tilde{F}}_{X}^{n+1},{\tilde{F}}_{Y}^{n+1},{\tilde{M}}_{Z}^{n+1}\right)$ denote the solution after and before under-relaxation at time level *n*+1, respectively.
4. Obtain the rigid whole-body velocity at time level *n*+1 by the structure Eq (9) with the computed hydrodynamic force and moment. A three-level second order scheme is employed for the derivative of velocity and this allows consistency with the second-order temporal discretization for the unsteady term we use in FLUENT.
  $$\{\begin{array}{l} \frac{3u_{TX}^{n+1}-4u_{TX}^{n}+u_{TX}^{n-1}}{2\Delta t}=\frac{F_{X}^{n+1}}{m}\\ \frac{3u_{TY}^{n+1}-4u_{TY}^{n}+u_{TY}^{n-1}}{2\Delta t}=\frac{F_{Y}^{n+1}}{m}\\ \frac{3\Omega_{Z}^{n+1}-4\Omega_{Z}^{n}+\Omega_{Z}^{n-1}}{2\Delta t}=\frac{M_{Z}^{n+1}-\Omega_{Z}^{n}{\left(dI_{Z}/dt\right)}^{n}}{I_{Z}^{n}} \end{array}.$$ (11)
  where Δ*t* is the time-step size (*T*/200). In the present work, the computational domain is a 9*L*(*X*)×3*L*(*Y*)×1.5*L*(*Z*) cuboid tank which is discretized with a grid including 3.42 million elements. The choice of the time-step and grid size is based on a sensitivity study, as to be presented in Section 3.1. Owing to the fact that the fish undergoes combined rigid whole-body and flexible deformation motions, the computational domain is divided into three zones, as shown in Fig 5. Zone 1, which contains the front part of fish body, undergoes the rigid whole-body motion without relative mesh movement between the front body and surrounding fluid cells [39]. The rear body and caudal fin in Zone 2 perform the resultant rigid and deforming motion, while the outer boundaries of Zone 2 undergo the rigid motion. During the motion (referred to Fig 6), the volume grid inside Zone 2 are regenerated and smoothed by the remeshing and smoothing approaches provided by FLUENT. We discretize Zone 1 and 2 with unstructured tetrahedral meshes (see Fig 6) since the grid style is quite fit for the wide variety of biological flow configurations and flexible enough to handle arbitrarily complex moving boundaries. Zone 3, which contains no the fish, is meshed with a hexahedral structured grid (see Fig 6) and moving with the rigid whole-body velocity. The above numerical strategy has successfully achieved the motion simulation of the complex 3D flexible fish, as shown by deformable mesh during one period in Fig 6.

#### 2.2.2 Performance metrics

In the current study, the thunniform fish swims along the negative *X* direction, and therefore the forward velocity *V*_*f*_ and the longitudinal force *F*_*l*_ can be given by

$$\{\begin{array}{l} V_{f}=-u_{TX}\\ F_{l}=-F_{X} \end{array}.$$ (12)

Based on the simulations, after the self-propelled swimmer achieves its steady status with zero mean forces, the forward velocity is oscillatory with time around a constant average value. This behavior is consistent with previous self-propelled investigations [29, 51] and the final mean value is called the cruising velocity *V*_*s*_.

The two non-dimensional parameters which characterize the swimming performance of a thunniform fish are the Reynolds number (*Re*) and the Strouhal number (*St*), which can be defined as [51, 74]

$$Re=\frac{\rho V_{s}L}{\mu },$$ (13)

$$St=\frac{2A_{p}f}{V_{s}},$$ (14)

where 2*A*_*p*_ denotes the maximum lateral excursion of the tail peduncle end.

The efficiency of the caudal fin (the main propulsor) is one of important performance metrics for the thunniform fish. Referred to previous studies [65, 75, 76] on oscillating foils/fins, the efficiency of the caudal fin during the cruising stage is given by

$$\eta =\frac{P_{out}}{P_{in}},$$ (15)

where *P*_*out*_ and *P*_*in*_ are the mean output power and the mean input power of the caudal fin, respectively. We compute them as follows:

$$\left\{ \begin{array}{l} P_{out}={\bar{F}}_{l}V_{s}\\ P_{in}=-\frac{1}{T}\int_{t}^{t+T}\int_{S_{f}}(F_{y}(t)\frac{dy_{f}(t)}{dt}+M_{z}(t)\frac{d\theta (t)}{dt})dSdt \end{array}\right.,$$ (16)

where ${\bar{F}}_{l}$ is the mean thrust generated by the caudal fin, *S*_*f*_ denotes the fin surface, *F*_*y*_ (*t*) and *M*_*z*_ (*t*) are the transverse force and yawing moment produced by the caudal fin, respectively, and [*t*, *t*+*T*] denotes one period at the steady swimming state.

The definition of the whole-fish efficiency for self-propelled swimming proplem is still ambiguous [23, 77]. Unlike the caudal fin providing the thrust, the mean longitudinal force of the whole body is zero at the steady swimming state, which brings difficulties for output power calculation. To deal with the issue, alternate definitions of the whole-fish efficiency have been proposed in the literature. One apporach is to compute the thrust (output) power by only considering the pressure force [73, 78]. The reason is that when the airfoil reaches its steady state velocity, the pressure force is equal to the viscous force. However as indicated by Borazjani and Sotiropoulos [23, 24], at some intants the viscous force contributes to the thrust while the pressure force the drag. Another alternative is to employ the ratio of the kinetic energy of the body forward motion over the total input energy in one priod. The definition was proposed by Kern & Koumoutsakos [45] and has been used in some subsequent investigations [35, 41]. Nevertheless, as the fish mass tends toward zero the efficiency also tends toward zero, and similarly the body with large mass would produce high efficiency. This is a drawback of this definition as shown in [43].

The definition of the whole-fish efficiency in the work is inspired on extensive discussions by Schultz and Webb [77]. They suggested that two practical swimming characteristics should be mainly concerned: the swimming velocity and the required input power, and the comparison basis among various kinds of fishes would then be propulsive parameters such as ‘‘miles per gallon”. Following the above idea, we define the whole-fish efficiency during the cruising stage as

$$\zeta =\frac{V_{s}}{P_{total}},$$ (17)

where *P*_*total*_ is the total mean input power for the deformable rear body and oscillating caudal fin and *ζ* has an actual physical interpretation, *i*.*e*., the energy required for swimming each unit distance.

## 3 Results and discussion

### 3.1 Sensitivity study and validation

**Fig 7:**
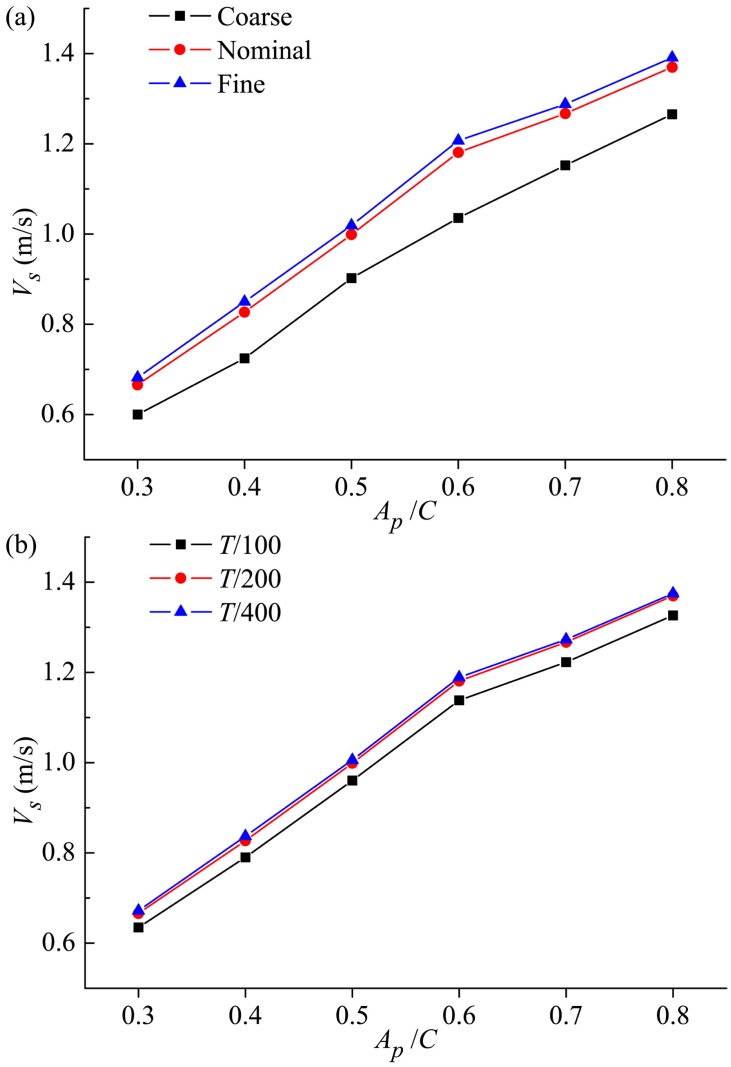
Grid and time-step sensitivity study for thunniform fish. Change on the crusing velocity with *A*_*p*_ for (a) three different grids and (b) three different time-step sizes.

Firstly, we present the nominal grid adopted in the current simulations. 3.42 million elements are used for the whole simulation domain. We employ the structured/unstructured hybrid grid technology, and zone the computational domain as inner region containing the fish and outer region. The inner region (namely Zone 1 and 2 in Fig 5) is discretized with unstructured grids, and the meshes are locally refined near the swimmer with element spacing size 0.005*L* to ensure sufficient resolution of the boundary layer. The proper transition of grid is ensured via properly controlling the grid size and growth rate from the body surface to the boundary of the inner region. An unstructured grid can better suit to the complex shape and movements of fish and reduce the time required for mesh dividing. On the other hand, in the outer region (namely Zone 3 in Fig 5), the mesh is rapidly stretched in directions of fish width and height. In the longitudinal direction, the grid stretching is rapid in the upstream region of the flexible body, while in the near wake region the stretching ratio of the mesh is kept below 5% so as to keep relatively high streamwise resolution. A structured grid is adopted in the outer region to decrease the grid number and improve the calculation efficiency. We have carried out comprehensive studies to assess the sensitivity of the numerical solution to the grid and time-step size. A grid sensitivity study is conducted using three meshes including 1.70 (coarse), 3.42 (nominal) and 6.98 (fine) million elements and a time-step size Δ*t* = *T*/200. The test is carried out with *θ*_*m*_ = 25°, *f* = 1.0Hz and *A*_*p*_/*C* = 0.3–0.8. The variations of the crusing velocity with *A*_*p*_ for three different grids are shown in Fig 7a. It can be seen that both the nominal and fine meshes generate the reasonable simulation results that are quite close to each other, while the coarse mesh cannot resolve the flow well. Therefore, the computed results based on the nominal grid are insensitive to further refinement of the grid size. A sensitivity study on Δ*t* is also carried out using the nominal grid and three different time-step sizes of *T*/100, *T*/200 and *T*/400. The resulting plot is given in Fig 7b where Δ*t* = *T*/200 proves to be suitable. Consequently, the setup with the nominal grid and the time-step size of *T*/200 allows a satisfactory computation of the FSI problem and corresponding dynamic response, and it is selected for all simulations of flow around the fish in this paper.

**Fig 8:**
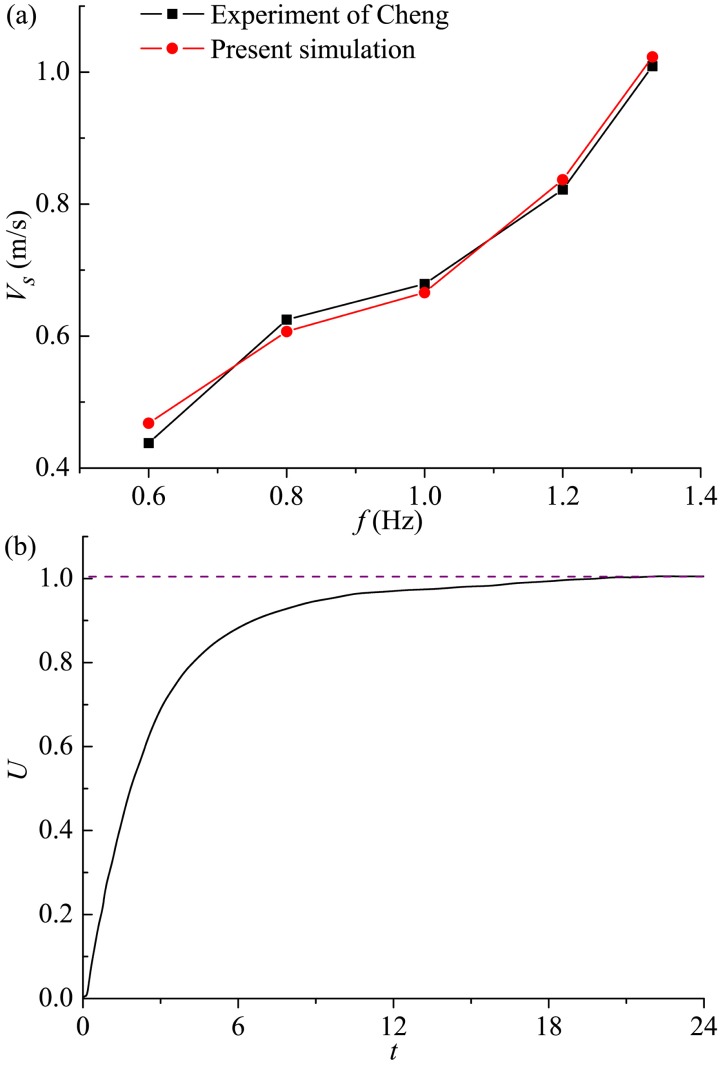
Validation of the numerical method. (a) Comparison of computed cruising velocity with established experimental results for different motion frequencies. (b) Falling speed of the sphere reaching an asymptotic value of 1.005.

In order to further validate the FSI method used to deal with the 3-DoF self-propelled swimming which is the subject of the work, we compare the computed cruising velocity with the experimental data of “*FangSheng-I*” [56], as shown in Fig 8a. Our simulation has employed the geometry of “*FangSheng-I*” as presented in Section 2.1. In terms of body kinematics, the experiment of Cheng [56] used the kinematics of a linked body, where the flexible rear body was segmented into five discrete links with the motion pattern described in Eq (3) for each link. Hence in the validation test, the number of links is set at *N* = 5 to match with the experiment. In order to reproduce the flow conditions of the experiment, the validation study is conducted for the body behavior parameter *A*_*p*_ = 0.3*C* and the caudal fin behavior parameter *θ*_*m*_ = 25° unchanged, and the variation of *V*_*s*_ with *f* obtained by our numerical approach matches well with experimental results of Cheng [56]. The implementation of the self-propelled framework is also validated by the test case of a falling sphere at *Re*_*d*_ = *ρ*_1_*Ud*/*μ*_1_ = 100. A rigid sphere with *ρ*_*s*_ (the sphere density) >*ρ*_1_ (the density of the fluid in which the sphere is immersed) is released from static state and accelerates until it attains its asymptotic falling speed. The diameter of the sphere *d* is taken as 1.0 and the density and dynamic viscosity of the fluid is chosen as *μ*_1_/*ρ*_1_ = 0.01 to aim for an asymptotic falling speed *U* = 1.0. The drag coefficient *C*_*D*_ for the flow around a sphere is 1.1 for *Re*_*d*_ = 100 (see Johnson and Patel [79]). In order to determine constant *g* and *ρ*_*s*_ of the test case, one writes *F*_*grav*_−*F*_*buoy*_ = *F*_*D*_ = *C*_*D*_[0.5*ρ*_1_*U*^2^(*πd*^2^/4)], and from this one gets *ρ*_*s*_/*ρ*_1_ = 1+*C*_*D*_(3*U*^2^/4*g*). We choose *g* = 20 and therefore obtain a sphere density of 1.041. We have used a 6.40×10^5^ non-uniform grid with relatively high resolution provided around this sphere and in the near wake. Additionally, the computational domain size employed is 15*d*×15*d*×15*d*. For comparison, Johnson and Patel [79] used a 101×42×101 body-conformal spherical grid on a domain of size 15*d*. Fig 8b shows the variation of the falling speed with time. At time *t* = 24 an asymptotic falling speed of 1.005 is attained, which matches well with the predicted value pertaining to the chosen parameters. For comparison, Kern and Koumoutsakos [45], who employed a grid with 4.00×10^5^ cells for the same test case, obtained an asymptotic falling speed of 1.006 at time *t* = 20.

### 3.2 Numerical analysis on accelerating-cruising process

To study variations of the kinematic characteristics, hydrodynamic performance and flow structure during the process where the fish accelerates from static status to stable cruise, we have conduceted simulations of several cycles to achieve the fully developed status resulting from the flexible fish kinematics. The swimmer starts from rest to an asymptotic constant velocity as the average thrust is compensated by the average drag.

#### 3.2.1 Kinematic characteristics

**Fig 9:**
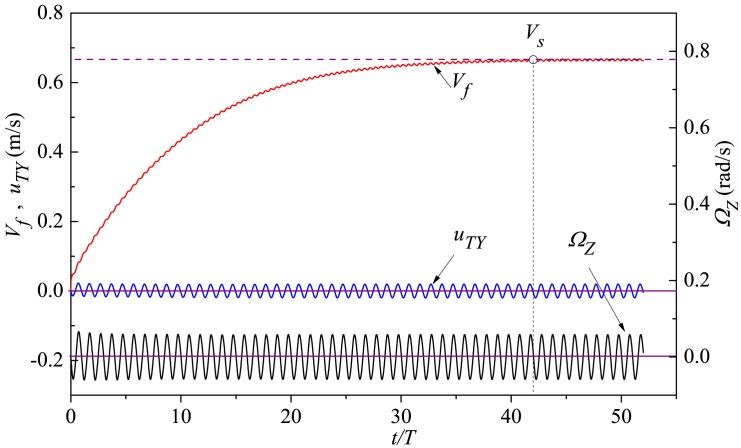
Temporal variation of forward velocity *V*_*f*_, transverse velocity *u*_*TY*_ and yawing angular velocity Ω_*Z*_. The cruising velocity *V*_*s*_ at the fully developed state is denoted by the horizontal dashed line. The balance positions of transverse velocity *u*_*TY*_ and yawing angular velocity Ω_*Z*_ are represented by the horizontal solid lines.

In the process where the fish accelerates from static state to steady cruise, the variations in the forward velocity *V*_*f*_, transverse velocity *u*_*TY*_ and yawing angular velocity Ω_*Z*_ with time are depicted in Fig 9. For this analysis, we focus on the case with *θ*_*m*_ = 25°, *A*_*p*_/*C* = 0.3 and *f* = 1.0Hz, and similar plots for other combinations of kinematic parameters (not provided here) show substantially all the qualitative trends. The thunniform swimmer accelerates from static state to an asymptotic mean forward velocity of *V*_*s*_ = 0.666 m/s (0.278 *L*/s) and oscillates with an amplitude of 0.0027 m/s (0.0011 *L*/s). The above qualitative characteristic is in line with other published studies on self-propelled swimming (Kern and Koumoutsakos [45], Borazjani and Sotiropoulos [29], Tytell *et al*. [62] and Gazzola *et al*. [80]). For instance in Kern and Koumoutsakos’s simulations [45], an anguilliform swimmer with reference motion pattern accelerated from rest to an asymptotic mean longitudinal velocity of *V*_*s*_ = 0.40 *L*/s and fluctuates with an amplitude of 0.01 *L*/s.

Since the current study is centered on the 3-DoF free-moving body, the fish performs not only forward movement like a number of past investigations on self-propulsion [29, 46, 50, 81, 82] but also transverse and yawing motions. As presented in Fig 9, the resultant whole-body kinematics of the thunniform fish includes periodically lateral translation and yawing rotation, with constant small amplitude and zero mean value. The above qualitative behaviors are consistent with numerical results of self-propelled anguilliform swimming reported by Kern and Koumoutsakos [45] where the transverse velocity has zero mean value and an amplitude of 0.03 *L*/s, and in experiments a thunniform swimmer under self-propulsion does show periodic stable translation and rotation as it performs forward locomotion on the whole [56].

#### 3.2.2 Hydrodynamic performance

**Fig 10:**
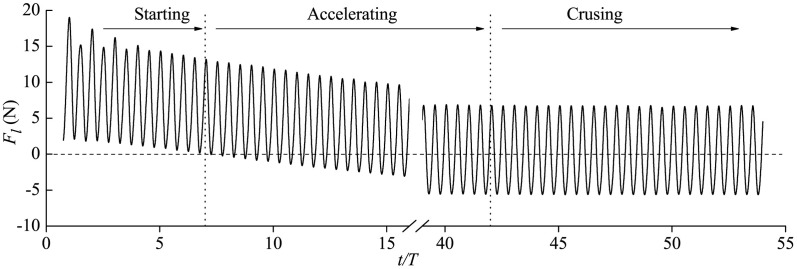
Time history of total longitudinal force *F*_*l*_.

For thunniform swimming under a self-propelled 3-DoF condition, the dynamical behaviors of the fish are purely determined by the complicated hydrodynamic forces. The total longitudinal force is shown in Fig 10 and converges to fluctuating modes with zero mean value at the crusing stage from thrust-type force at the starting and accelerating stages.

**Fig 11:**
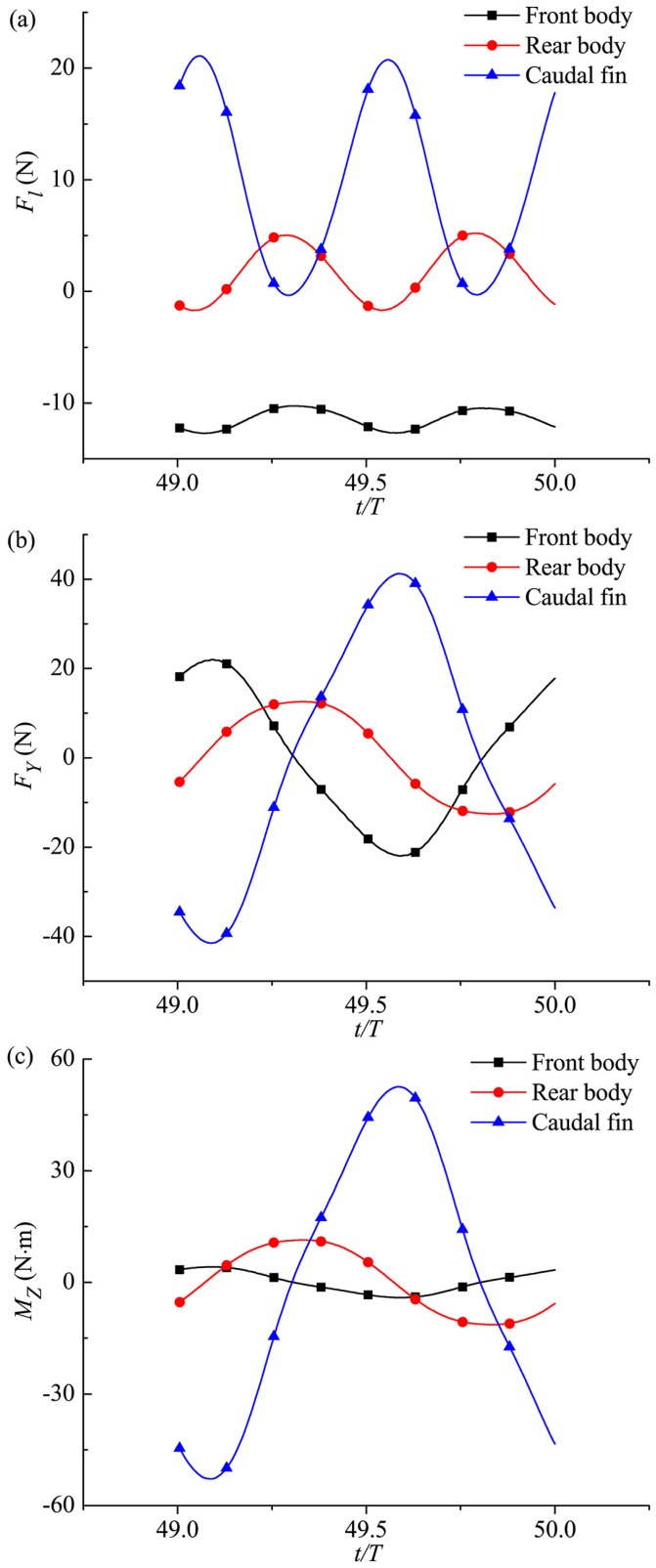
Fluid forces (moments) of front body, rear body and caudal fin during cruising in one specific cruising period. (a) Longitudinal force, (b) transverse force and (c) yawing moment.

Hydrodynamic analysis on different parts of fish body at the crusing stage is presented as follows. Fig 11a shows that *F*_*l*_ of all these body parts is in fluctuation during one period with two peaks. In one swimming cycle the longitudinal force of the front body is always of drag type, and however the *F*_*l*_ of the rear body with flexible deformation is of thrust type in a certain amount of time within one period while of drag type at other times. The caudal fin, providing most of the thrust, is the main propulsor of fish swimming. The finding that not exclusively the caudal fin produces thrust but also the remaining moving part of the body is in agreement with the numerical results of van Rees *et al*. [48, 83] for anguilliform swimming. We now move on to the transverse force *F*_*Y*_ and the yawing moment *M*_*Z*_ in Fig 11b and 11c, and *F*_*Y*_ and *M*_*Z*_ of all fish body parts are observed with fluctuating variation with single peak during each cycle. Fluctuation amplitudes of *F*_*Y*_ and *M*_*Z*_ of caudal fin are in maximum value among all these body parts.

#### 3.2.3 Flow structure

**Fig 12:**
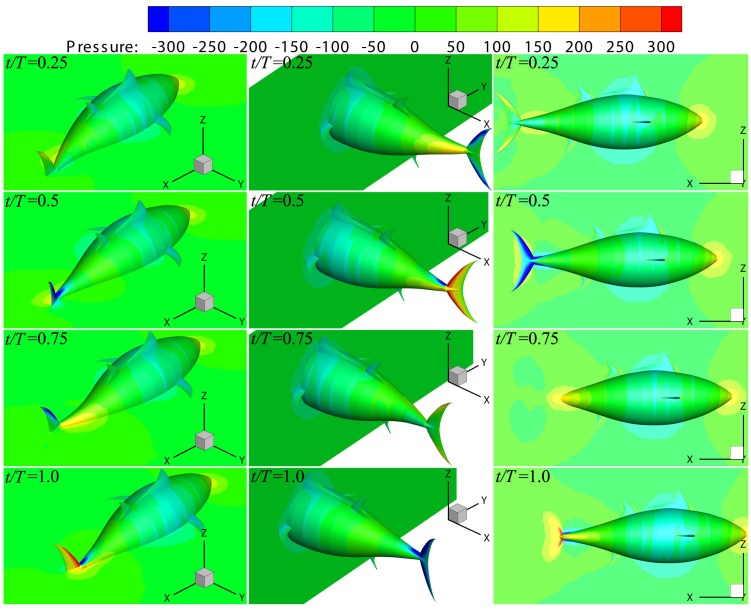
Pressure contours on the fish body and selected sectional planes at four instants during one specific cycle at the cruising stage. The color scheme for pressure contours is such that red colors denote the highest pressure and blue the lowest pressure.

To understand the propulsion mechanism of thunniform swimmers, in this section we first analyze the variation of pressure in the flow field during cruising, which is based on Fig 12. Note that the pressure itself is not the solution of Navier-Stokes equation, and pressure difference has to be considered instead. For each phase, three plots are shown in Fig 12. The left-hand-side plot presents a perspective view of pressure contours on the body surface and the mid-depth plane, and the middle plot presents the body surface and the sectional cut parallel to the *yoz* plane at about quarter position of the body length. The right-hand-side plot shows a side view of pressure contours on the body surface and the mid-width plane.

It can be seen from Fig 12 that during one cycle, the pressure experienced by the caudal fin changes with time in the form of positive-negative symmetry. In particular, at *t*/*T* = 0.5 (the second row of Fig 12), a large low pressure region emerges at the right-side surface of the caudal fin (viewed from the tail), and a large high pressure area is created at the left-side surface. Thus, there is a large pressure difference on both sides, leading to the thrust, transverse force and yawing moment of the caudal fin almost attaining the peak value (referred to Fig 11). Although the pressure distribution situation of the tail at *t*/*T* = 1.0 (the fourth row of Fig 12) is opposite to that at *t*/*T* = 0.5, however the oscillating angle of caudal fin at this moment is also opposite to that when *t*/*T* = 0.5 (referred to Eq (2)), so the thrust of caudal fin is almost at the peak as well (see Fig 11a). The variation of pressure on the rear body over time is similar to that on the caudal fin, but the amplitude is much smaller. However, the variation of pressure on the surface of the front body and flow field close by is slight during one period, and therefore the drag, transverse force and yawing moment experienced by the front body show small fluctuation (referred to Fig 11).

**Fig 13:**
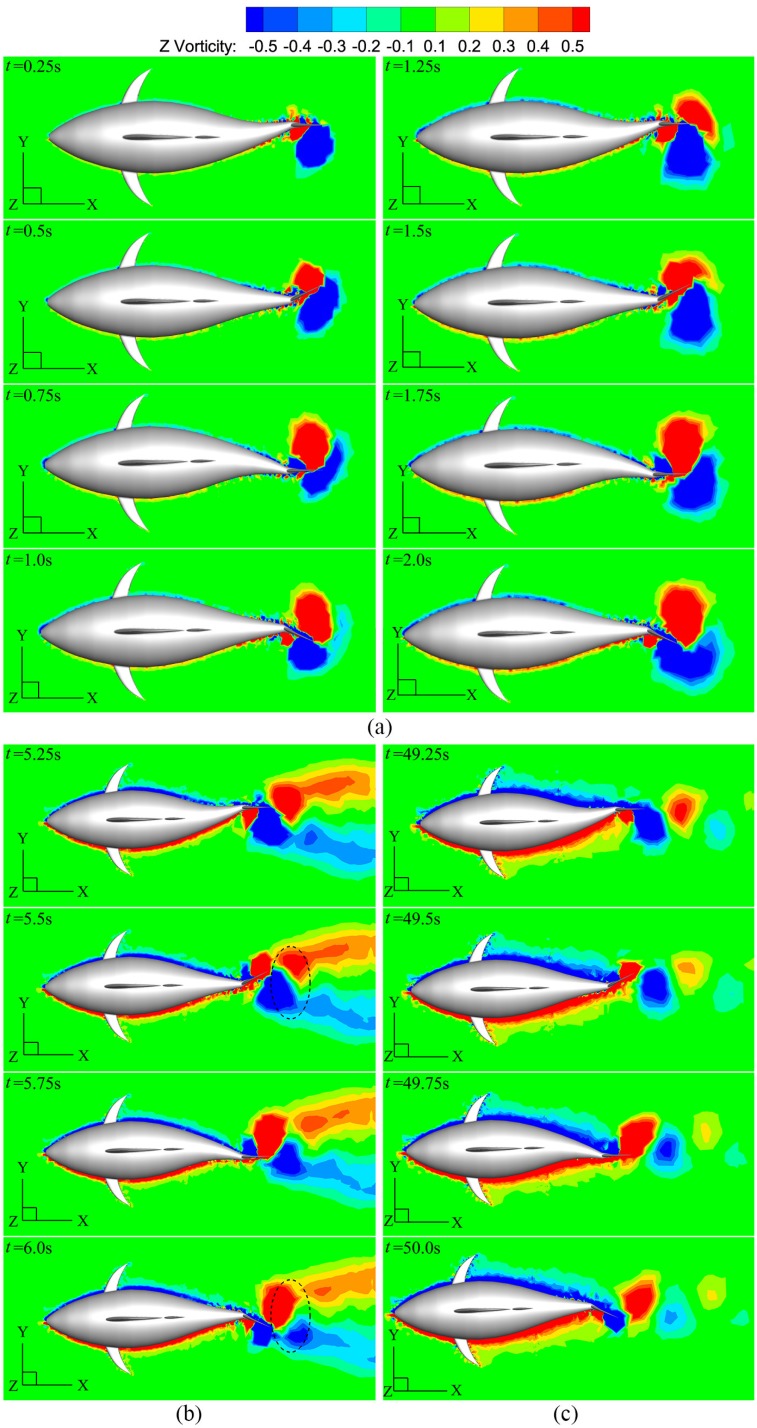
Vorticity contours for different swimming stages. (a) Starting stage, (b) accelerating stage and (c) cruising stage.

Fig 13 shows the instantaneous vorticity field on the mid-depth plane from impulsive start to steady cruise. During start process (Fig 13a), the tail generates two counter-rotating vortices in one circle. As swimming speed is slow and swimming distance short, the wake vortices within the same cycle would push each other and the same sign vortices of two consecutive cycles would merge. Once the fish starts to swim forward, a transitional stage is needed before it finally achieves a stable state. Interestingly, when the vorticity convects downstream, a vortex dipole is observed (as the dashed circle in Fig 13b where the red vortex corresponds to a counterclockwise one and the blue a clockwise one). It carries asymmetric suction force between left-side surface and right-side surface of the caudal fin, leading to continuously increasing forward velocity, and this will soon become clear. Finally, a steady swimming state is achieved as the wake being a reverse Kármán vortex street consisting of a single row of vortices (Fig 13c), which agrees well with previous investigations on thunniform swimmers [2, 10, 51, 84]. Additionally, the caudal-fin-generated vorticity merges with the same-sign body-generated vorticity to produce one vortex structure as the tail tip varies motion direction every half period, leading to a strong and well-organized vortex street downstream. The interaction mode is similar to the enhancing interference in the study of Triantafyllou *et al*. [85] on the foil-vortex interaction and the constructive mode mentioned by Zhu *et al*. [10] in fish-like swimming.

**Fig 14:**
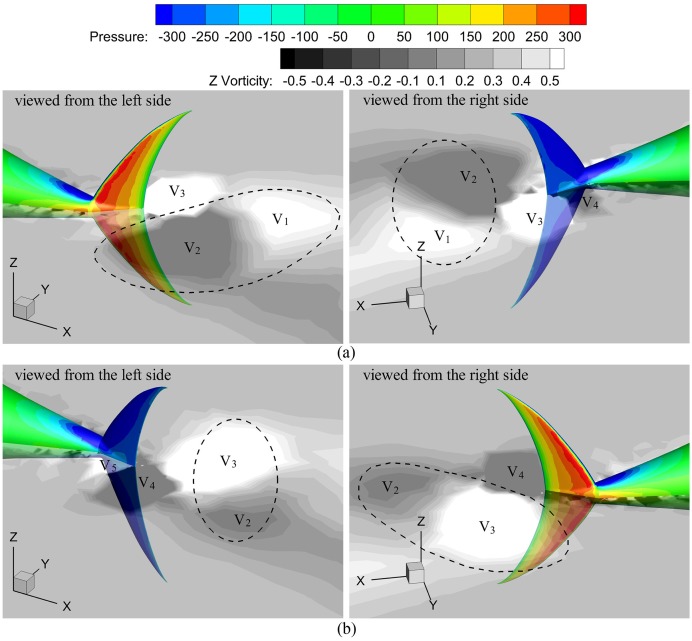
Pressure distribution of the tail with mid-depth-plane vorticity field during accelerating stage. (a) *t* = 5.5s and (b) *t* = 6.0s.

It is necessary to explain the vortex dipole observed in Fig 13b and its relation with asymmetric suction force, which is based on Fig 14. This figure presents the vorticity field on the mid-depth plane and the pressure distribution of the tail at two instants during accelerating stage. For each intant, two plots from different views are shown. In Fig 14a, vortex V_2_ have completely shed from the trailing edge of the caudal fin, pairing up with vortex V_1_ shed during the preceding half cycle to form a vortex dipole (as the dashed circle). A large low pressure region is created at the right-side surface (suction surface) of the fin due to attached vortices V_3_ and V_4_. After half period (referred to Fig 14b), with the complete shedding of vortex V_3_, a new vortex dipole is produced (as the dashed circle). Under the impact of attached vortices V_4_ and V_5_, the left-side surface of the fin becomes suction surface. Consequently, vortices are shed in alternating order from the fin trailing-edge and tend to pair with the counterparts of opposite sign to form vortex dipoles. During the process, the suction surface of the fin changes in alternating order, and the asymmetric suction force at left and right sides of the fin is carried by vortex dipoles.

### 3.3 Effect of the tail undulating amplitude

In this section, we study the effect of the undulating amplitude of the tail peduncle end. For this analysis, *A*_*p*_ is adjusted in small steps from 0.3*C* (0.043*L*) to 0.8*C* (0.113*L*), which covers the range of 0.052–0.082*L* reported in Donley and Dickson’s experiment [53].

#### 3.3.1 Forward velocity and longitudinal force

**Fig 15:**
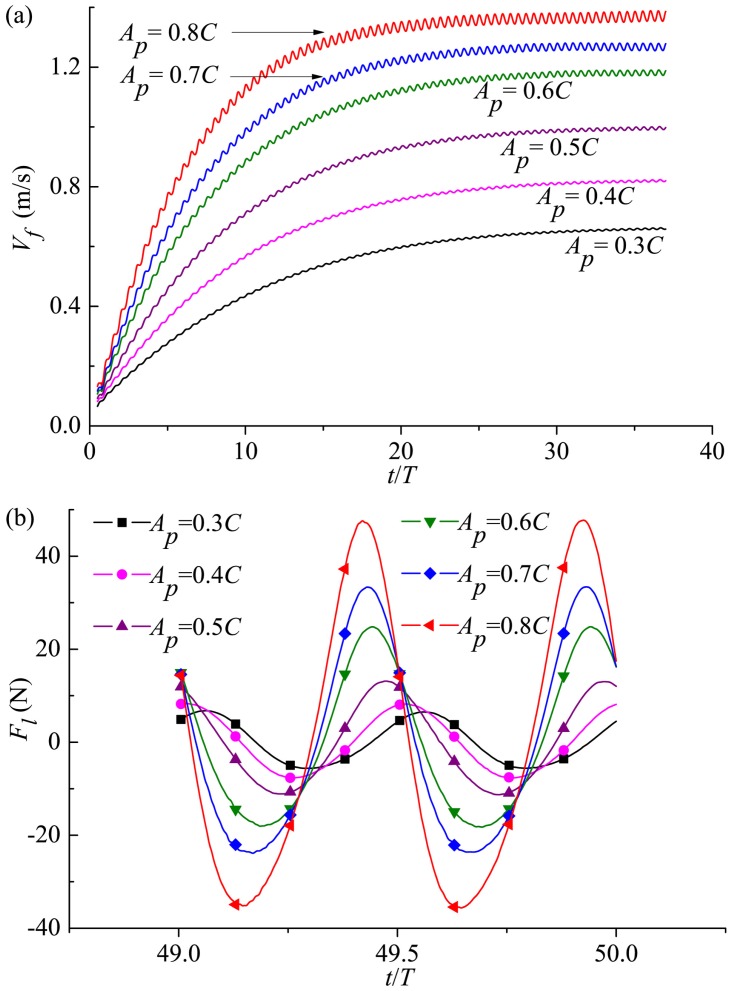
Variation in (a) forward velocity and (b) longitudinal force with time for various *A*_*p*_. For all these cases, *θ*_*m*_ = 25° and *f* = 1.0Hz.

During the self-propelled process, the time variations of the forward velocity and longitudinal force for different tail undulating amplitudes are shown in Fig 15. It is clear from Fig 15a that, the cruising velocity becomes larger with the increase of *A*_*p*_. In fact, 106% more *V*_*s*_ is obtained as *A*_*p*_ increases from 0.3*C* to 0.8*C* based on the current computations, and the Reynolds number appears within the interval 1.59×10^6^–3.27×10^6^. For comparison, the median *Re* value reported in Dewar and Graham’s experiment [8] on tropical tunas is 2.01×10^5^, and the median *Re* value of bluefin tunas reported by Wardle *et al*. [52] is 4.68×10^6^. Therefore, the flow regime of typical thunniform swimmers is tackled in our study, which will be used here as the biological reference to place the simulations into context. In Fig 15b for all examined *A*_*p*_, the longitudinal force in one specific period during cruising shows two peaks that correspond to the caudal fin strokes at both sides. The observation is in line with previous studies on carangiform swimming and anguilliform swimming of Borazjani and Sotiropoulos [23, 24] and thunniform swimming of Xia *et al*. [51]. Furthermore, the peak value of *F*_*l*_ increases monotonically with the undulating amplitude of the tail peduncle end.

#### 3.3.2 Transverse force and yawing moment

**Fig 16:**
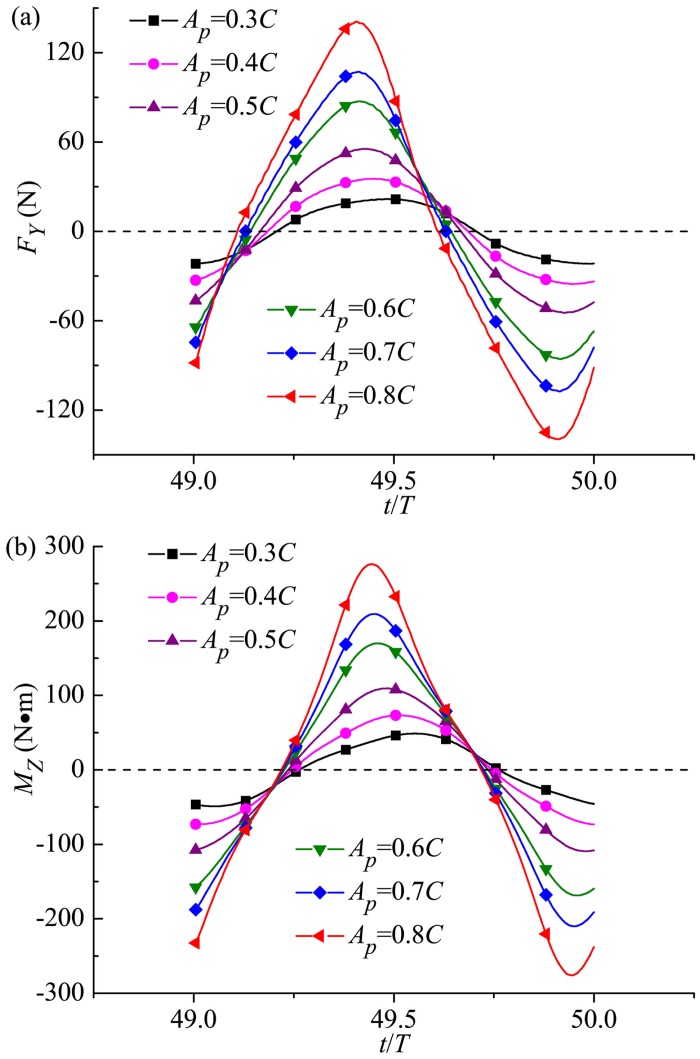
Temporal variation of (a) transverse force and (b) yawing moment for various *A*_*p*_. For all these cases, *θ*_*m*_ = 25° and *f* = 1.0Hz.

The transverse force and yawing moment in one period during cruising with respect to *A*_*p*_ is shown in Fig 16. Because of the symmetry of undulating locomotion, the mean transverse force and yawing moment is zero during one cycle. Additionally, the increasing amplitude values of *F*_*Y*_ and *M*_*Z*_ are observed as the undulating amplitude of the tail peduncle end increases. The simulation result implies that the case with *A*_*p*_ = 0.8*C* produces the largest transverse disturbance on the fluid, and more power is wasted in the transverse direction rather than in the longitudinal direction (to generate propulsive power in the *X* direction). Gazzola *et al*. [86] have uncovered a unifying mechanistic principle characterizing aquatic locomotion, can be simply couched as the power law *Re*~*Sw*^*α*^, where *Sw* = 2*A*_*p*_*ωL*/*ν* (*ω* = 2*πf*), with *α* = 4/3 for laminar flows, and *α* = 1 for turbulent flows. In the current turbulent simulations, the dimensionless number characterizing the undulatory motions *Sw* ranges between 3.06×10^6^ and 8.16×10^6^, and our results follow the above power law for turbulent flows.

#### 3.3.3 Power, efficiency and wake structure

In order to study the effect of *A*_*p*_ on the power requirement, various input power metrics are given in Table 1 for different tail undulating amplitudes. Simulation results clearly show that the average input power of the whole fish *P*_*total*_, the average input power of rear body *P*_*r*_ and the average input power of caudal fin *P*_*in*_ monotonically increases with *A*_*p*_. It is important to point out that the increase in cruising velocity at larger *A*_*p*_ (see Fig 15a) is not for free, since the swimmer has to beat its tail peduncle and caudal fin with larger transverse excursion to reach the faster speed and more power is therefore consumed to achieve this.

**Table 1:** Power requirement for various tail undulating amplitudes.

*A*_*p*_/*C*	*P*_*total*_ (W)	*P*_*r*_ (W)	*P*_*in*_ (W)
0.3	15.91	0.92	14.99
0.4	28.26	2.11	26.15
0.5	48.33	4.25	44.08
0.6	83.98	8.54	75.44
0.7	108.99	12.16	96.83
0.8	152.04	18.51	133.53

**Fig 17:**
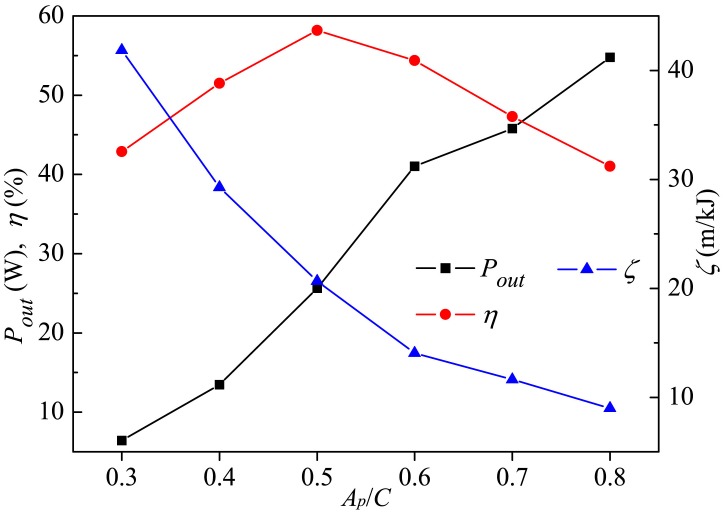
Variation in output power and efficiency of caudal fin and the whole-fish efficiency with *A*_*p*_. For all these cases, *θ*_*m*_ = 25° and *f* = 1.0Hz.

Fig 17 shows the output power and efficiency of the caudal fin together with the whole-fish efficiency as functions of *A*_*p*_. It is interesting to note that in spite of the monotonical increase in fin output power *P*_*out*_ with *A*_*p*_, the efficiency of the caudal fin *η* initially increases as *A*_*p*_ is rising with a critical point of 0.5*C* and then decreases as *A*_*p*_ is further increased. On the other hand, the whole-fish efficiency *ζ* decreases with increasing *A*_*p*_. It should be mentioned that, since the body gait is responsible for the caudal fin’s swaying motion and the fin's own rotation is responsible for its oscillating motion, not only the flow prepared for the fin by the body changes but also the fin no longer maintains the same kinematics as the body gait *A*_*p*_ is changed, and the variation of *η* results from the above two factors.

**Fig 18:**
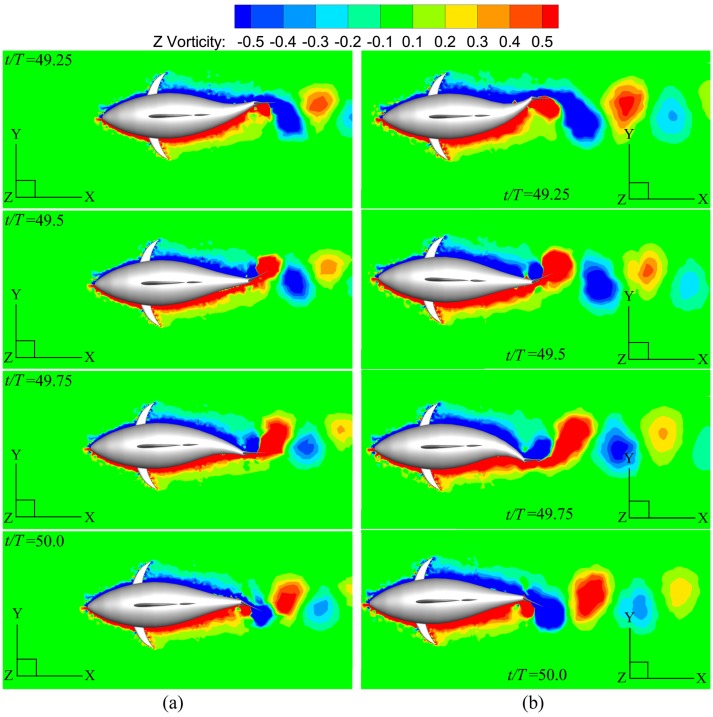
Vorticity field at four discrete phases during one period for various *A*_*p*_. For all these cases, *θ*_*m*_ = 25° and *f* = 1.0Hz. (a) *A*_*p*_ = 0.4*C* and (b) *A*_*p*_ = 0.6*C*.

**Fig 19:**
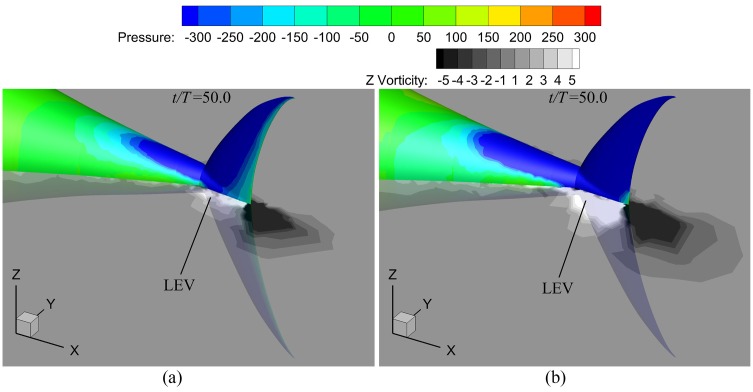
Mid-depth-plane vorticity contours with pressure distribution of the tail for various *A*_*p*_. For all these cases, *θ*_*m*_ = 25° and *f* = 1.0Hz. (a) *A*_*p*_ = 0.4*C* and (b) *A*_*p*_ = 0.6*C*.

At the steady swimming state, the instantaneous vorticity contours on the mid-depth plane for different *A*_*p*_ are depicted in Fig 18. The single row vortex street is seen with similar arrangement form for all these cases. In Fig 18 small vorticity level is used to identify reverse Kármán vortex street in its completeness, and therefore we cannot avoid losing the details of the vortices in those regions where vorticity is large (*e*.*g*. near the tail). In order to clearly explain the connection between the wake structure and swimming performance, we plot Fig 19 to highlight the difference in vortex strength for different *A*_*p*_ by increasing the displayed vorticity level. Now clearly to see is that the area occupied by high-vorticity region in leading-edge vortex (LEV) significantly increases with the tail undulating amplitude, leading to the increase of the area of suction region on the caudal fin. Hence, the fin will generate larger thrust as *A*_*p*_ is raised (based on the current simulations 113% more thrust is generated by the fin as *A*_*p*_ is varied from 0.4*C* to 0.6*C*), and the fish will swim faster so as to make the fin thrust is balanced by the body drag (referred to Fig 15).

**Fig 20:**
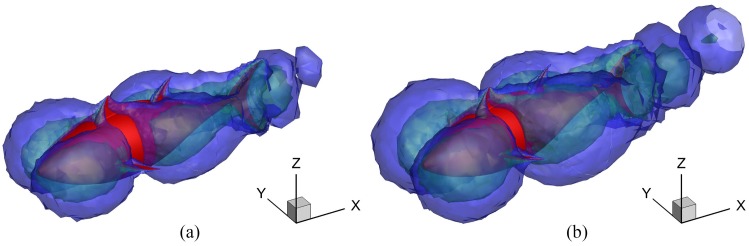
Iso-surface of the square of velocity in the transverse direction for different *A*_*p*_. For all these cases, *θ*_*m*_ = 25° and *f* = 1.0Hz. The blue region means |*u*_*TY*_| = 0.05 m/s; the green region means |*u*_*TY*_| = 0.1 m/s and the fish is drawn in red. (a) *A*_*p*_ = 0.4*C* and (b) *A*_*p*_ = 0.6*C*.

The square of the transverse velocity *u*_*TY*_ in the flow field actually indicates the power wasted by undulating locomotion (the energy loss in the transverse direction). The iso-surfaces of (*u*_*TY*_)^2^ for differdent *A*_*p*_ are presented in Fig 20. For the case with larger *A*_*p*_, because of the larger transverse disturbance on the fluid, more power is wasted in the transverse direction, which indicates the lower efficiency *ζ*.

### 3.4 Effect of the oscillating amplitude of the caudal fin

In the above section, in order to examine the effect of body behavior parameter, we systematically vary *A*_*p*_ to analyze the swimming performance and wake structure. In addition to body kinematic behaviors, caudal fin behavior parameters are also expected to play an important role, and therefore we now move on to the effect of the oscillating amplitude of the caudal fin. Herein *θ*_*m*_ is changed in the range of 15°–35°, and as a comparison, in the study of Akhtar *et al*. [87] the oscillating amplitude was between 20° and 30° and Yang *et al*. [11] used *θ*_*m*_ of 5°–30° to investigate the propulsion mechanism for oscillating tuna-tails.

#### 3.4.1 Forward velocity and longitudinal force

**Fig 21:**
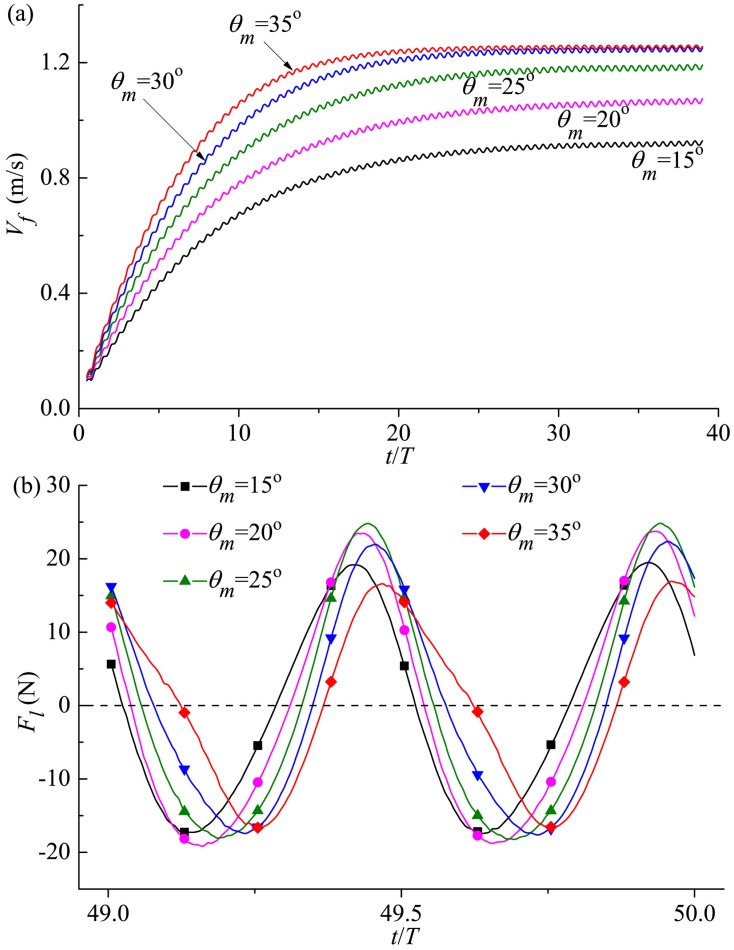
Time history of (a) forward velocity and (b) longitudinal force for different oscillating amplitudes of the caudal fin. For all these cases, *A*_*p*_/*C* = 0.6 and *f* = 1.0Hz.

During accelerating-cruising process, the variations of the forward velocity and longitudinal force with time for different *θ*_*m*_ are plotted in Fig 21. When *θ*_*m*_≤30° the cruising velocity increases obviously with the augmentation of the oscillating amplitude in Fig 21a, and the present computations show the swimmer obtains 34% more *V*_*s*_ as *θ*_*m*_ is changed from 15° to 30°. However, *V*_*s*_ when *θ*_*m*_ = 35° is very close to that when *θ*_*m*_ = 30°, indicating that continuing to raise the oscillating amplitude has quite limited effect on the increase of *V*_*s*_. In the current study, *Re* ranges between 2.20×10^6^ and 3.00×10^6^ and matches with the experimental flow regime of typical thunniform fish (Dewar and Graham [8] and Wardle *et al*. [52]). It can be seen from Fig 21b that, although the longitudinal force during one specific cycle at the cruising stage shows periodic fluctuation with two peaks for all cases, the time for *F*_*l*_ to reach the crest delays with the increase of *θ*_*m*_. Furthermore, the peak value of *F*_*l*_ increases firstly as *θ*_*m*_ is raised from 15° to 35° with a turning point of 25° and then decreases as *θ*_*m*_ is further increased.

#### 3.4.2 Transverse force and yawing moment

**Fig 22:**
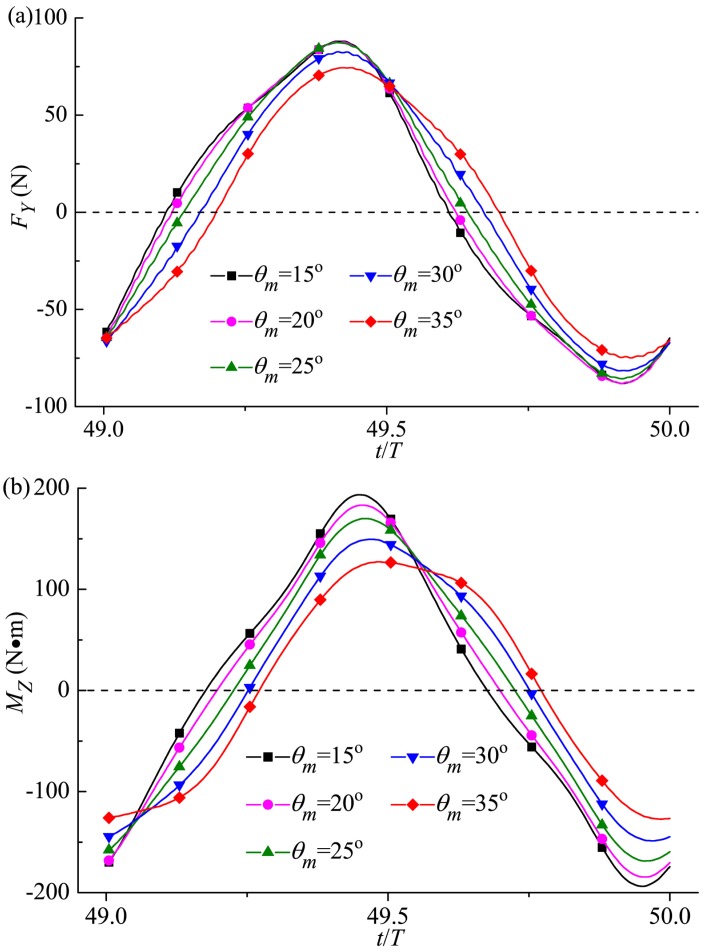
Variation in (a) transverse force and (b) yawing moment with time for various oscillating amplitudes of the caudal fin. For all these cases, *A*_*p*_/*C* = 0.6 and *f* = 1.0Hz.

Fig 22 presents the transverse force and yawing moment in one cruising cycle for various *θ*_*m*_. Generally, with the increase of the oscillating amplitude, the amplitude values of *F*_*Y*_ and *M*_*Z*_ go down and the fluctuation phases get retarded. It should be noted that the relation between the dimensionless number characterizing the transverse undulation *Sw* and the Reynolds number *Re* follows Gazzola *et al*.’s scaling law of aquatic swimming [86]. Following Gazzola *et al*. [86], we have *Re*~*Sw*. Currently, *Re* in this simulation is in the order of magnitude of 10^6^, and the corresponding *Sw* is 6.12×10^6^. Additionally, the values for the maximum lateral excursion at the tip of the snout obtained in our simulations (2.53–4.55%*L* for the studied range of *θ*_*m*_) coincide with the range of 2.3–4.7%*L* observed for kawakawa tunas (Donley and Dickson [53]).

#### 3.4.3 Power, efficiency and wake structure

In this section we firstly investigate the effect of *θ*_*m*_ on the power requirement, which is based on Table 2 where various input power metrics are given for different oscillating amplitudes of the caudal fin. It is observed that *P*_*in*_ of caudal fin decreases gradually with increasing *θ*_*m*_ while *P*_*r*_ of rear body has little change, and therefore the total mean input power reduces. Combining the simulation results of forward velocity (see Fig 21a), it is easy to find out that the fish can achieve high-speed swimming accompanied by low power requirement by such kinematic mode.

**Table 2:** Power requirement for different *θ*_*m*_.

*θ*_*m*_ (°)	*P*_*total*_ (W)	*P*_*r*_ (W)	*P*_*in*_ (W)
15	84.88	8.13	76.75
20	84.11	8.46	75.65
25	83.98	8.54	75.44
30	83.03	8.55	74.48
35	80.87	8.35	72.52

**Fig 23:**
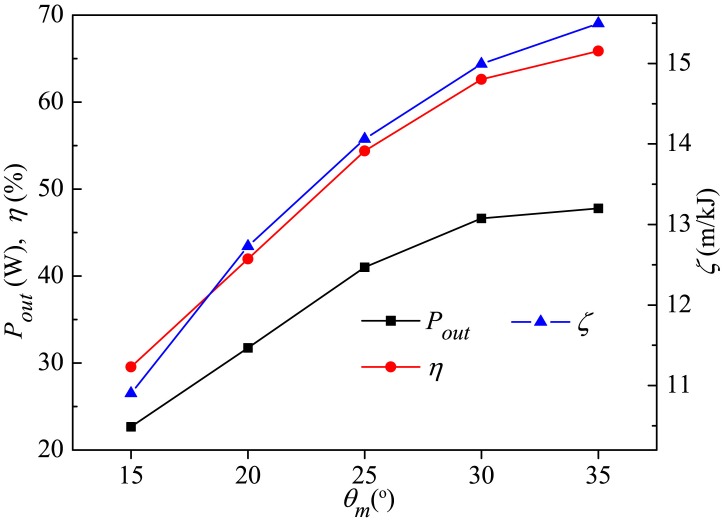
Variation in output power and efficiency of caudal fin and the whole-fish efficiency with *θ*_*m*_. For all these cases, *A*_*p*_/*C* = 0.6 and *f* = 1.0Hz.

The output power (*P*_*out*_) and efficiency (*η*) of the caudal fin and the whole-fish efficiency (*ζ*) for various *θ*_*m*_ are plotted in Fig 23. As the oscillating amplitude becomes larger, *P*_*out*_ of the caudal fin keeps increasing, and a gradually increasing *η* and *ζ* is observed. The Strouhal number in the present work is a decreasing function of *θ*_*m*_, and approaches the universal optimal value of 0.3 identified for efficient oscillatory propulsion of swimming animals (Triantafyllou *et al*. [60, 85]) as the efficiency of the caudal fin reaches the maximum value 66% at *θ*_*m*_ = 35°. Therefore by comparing the computed results in Fig 23 with Fig 21a, it can be concluded that a proper rise of oscillating amplitude can endow the thunniform fish with better swimming performance with the simultaneously increased velocity and efficiency.

**Fig 24:**
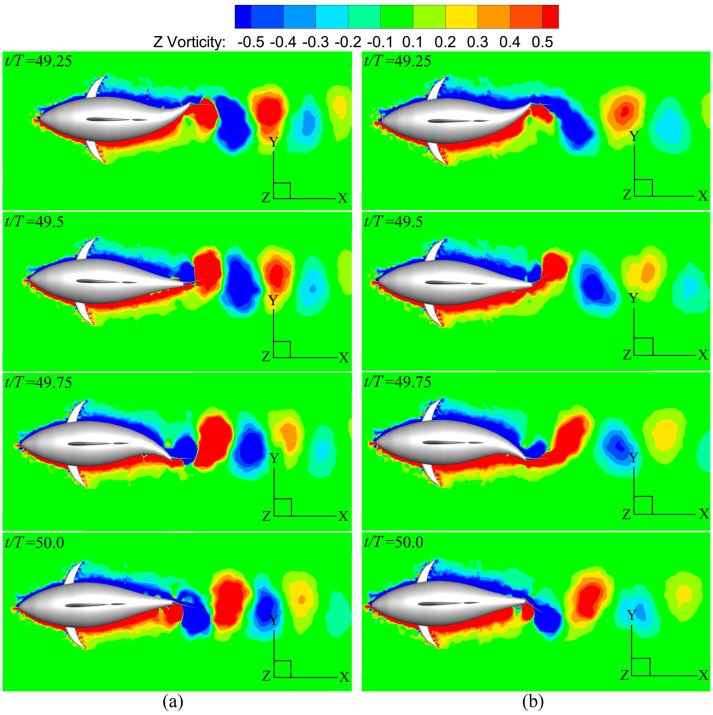
Vorticity contours at four instants in one period for different oscillating amplitudes of the caudal fin. For all these cases, *A*_*p*_/*C* = 0.6 and *f* = 1.0Hz. (a) *θ*_*m*_ = 15° and (b) *θ*_*m*_ = 35°.

**Fig 25:**
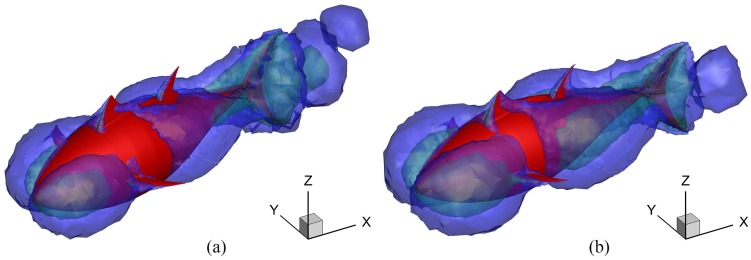
Iso-surface of the square of the transverse velocity for different *θ*_*m*_. For all these cases, *A*_*p*_/*C* = 0.6 and *f* = 1.0Hz. The blue region means |*u*_*TY*_| = 0.1 m/s; the green region means |*u*_*TY*_| = 0.2 m/s and the fish is drawn in red. (a) *θ*_*m*_ = 15° and (b) *θ*_*m*_ = 35°.

Fig 24 presents the instantaneous vorticity field on the mid-depth plane for *θ*_*m*_ = 15° and 35° during the cruising stage. For the lower-oscillating-amplitude case, the centers of the vortices are well aligned in the downstream direction. However for the higher-*θ*_*m*_ case, the centers of the vortices oscillate around the wake centerline. Since each pair of opposite sign vortices (a vortex dipole) induces a jet, the discrepancy in arrangement form of the vortex street implies that a stronger transverse flow will be generated at lower *θ*_*m*_. This becomes clear in Fig 25 where the iso-surfaces of the square of velocity in the transverse direction for different *θ*_*m*_ are shown. The smaller transverse disturbance on the fluid induced by fish swimming is observed for the higher-*θ*_*m*_ case. Thus, the kinetic energy loss in the wake is decreasing with increasing *θ*_*m*_; consequently, the power requirement also decreases.

**Fig 26:**
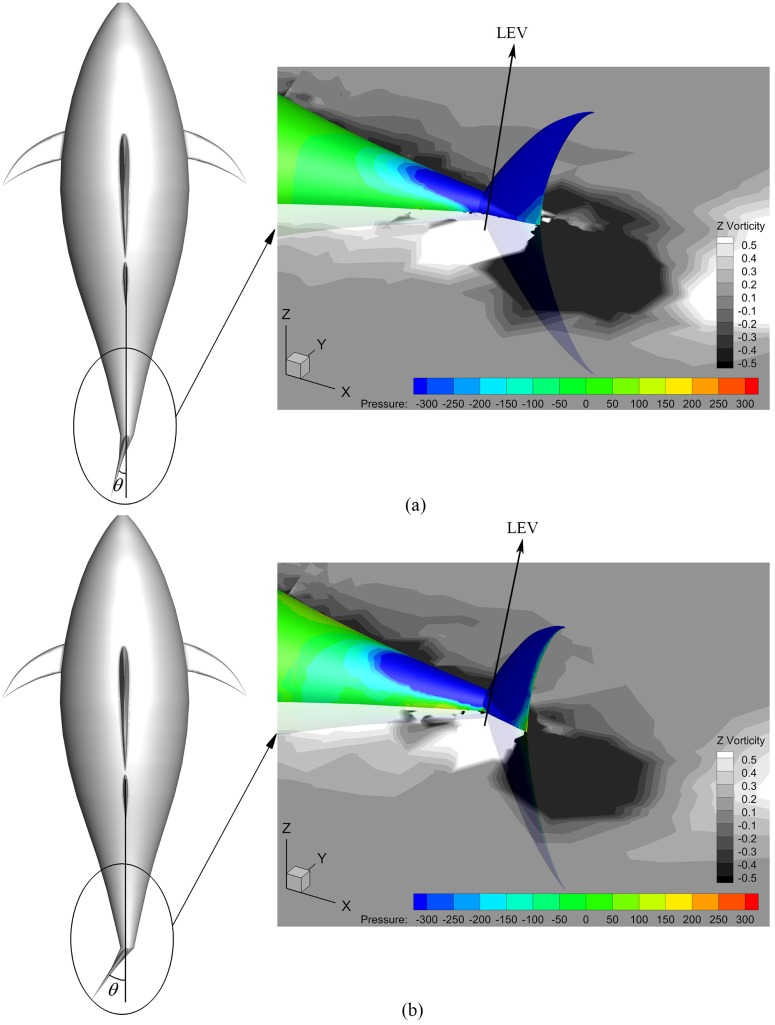
Fish postures with pressure distribution of the tail and mid-depth-plane vorticity field for various *θ*_*m*_. For all these cases, *A*_*p*_/*C* = 0.6 and *f* = 1.0Hz. (a) *θ*_*m*_ = 15° and (b) *θ*_*m*_ = 35°.

On the other hand, at the end of the tail beat in one cycle, an evident lower-pressure region in Fig 26 is observed on the caudal fin. Simulation results show that the area of suction region is more or less similar to each other, and the difference between smaller and larger amount of vorticity is marginal. However, the tail postures in the left-hand-side plots in Fig 26 demonstrate that the caudal fin is favorably inclined so that the suction induced by vortices would contribute to thrust generation (actually the fin thrust increases by a factor of about 1.56 as *θ*_*m*_ is raised from 15° to 35° according to the simulations). Thus, the thunniform swimmer can benefit more efficiently from leading-edge vortex (LEV) with the increase of *θ*_*m*_.

## 4 Conclusions

Numerical simulations are carried out to study the hydronamics of a thunniform swimmer which is undulated laterally in the viscous fluid and moved freely under a self-propelled 3-DoF condition. The complex interaction of the fish with surrounding viscous flow is achieved by the FSI method with an in-house developed UDF.

The computational results show that during the process of accelerating-cruising, the forward velocity increases gradually and eventually fluctuates with slight amplitude around *V*_*s*_ as the mean longitudinal force is zero. This qualitative characteristic is in agreement with previous investigations on self-propelled locomotion (Kern and Koumoutsakos [45], Borazjani and Sotiropoulos [29], Tytell *et al*. [62] and Gazzola *et al*. [80]). Hydrodynamic analysis on different parts of fish body shows that the caudal fin is a major source of thrust production, and interestingly the deforming rear body can also provide a small thrust in a certain period of time, which is similar to that found in anguilliform swimmers (van Rees *et al*. [48, 83]). The unsteady evolution process of the flow structure follows the vortex structures arranged almost vertical to the swimming direction with squeezing and merging of vortices, vortex dipole formation eventually leading to the propulsion motion, and finally a reverse Kármán vortex street. The simulation results clarify the wake structure as a single row of vortices observed for thunniform swimmers in previous experimental and numerical studies (Anderson [84], Zhu *et al*. [10], Yang and Su [2] and Xia *et al*. [51]).

A parametric study of the 3-DoF self-propulsion is conducted so as to discuss the effect of the body behavior parameter. The cruising velocity increases with *A*_*p*_, and the Reynolds number obtained in our simulations coincides with the flow regime documented in thunniform swimming experiments (Dewar and Graham [8] and Wardle *et al*. [52]). As *A*_*p*_ is raised, *F*_*Y*_ and *M*_*Z*_ vibrate with amplified amplitude and zero time-averaged value and the inspection on transverse velocity in the wake also indicates the larger transverse disturbance on the fluid. Hence more power is wasted in the transverse direction and the efficiency *ζ* decreases with increasing *A*_*p*_. Moreover, our computational results are supported by the principal scaling law of undulatory locomotion reported by Gazzola *et al*. [86]. Since both cruising velocity and power requirement monotonously increase with *A*_*p*_, the realization of high-speed swimming at larger *A*_*p*_ is not for free for the swimmer but it have to stroke with larger transverse excursion at the cost of more power consumption. This simulation result helps to explain Donley and Dickson’s observation [53], where kawakawa tunas prefer to raise their motion frequency rather than increase tail undulating amplitude in order to increase their swimming speed. Additionally, the Strouhal number obtained in this study (between 0.31 and 0.40) lies within the narrow range of *St* for oscillating foils of high propulsive efficiency (Triantafyllou *et al*. [88], Anderson *et al*. [75] and Read *et al*. [89]) and the interval of 0.2–0.4 identified for the efficient cruise of flying and swimming animals driven by their wing or tail (Taylor *et al*. [90]). The variation in the swimming performance (the fin thrust and cruising velocity) due to the increase of *A*_*p*_ is associated with the strength of vortices in the wake and the area of suction region on the caudal fin.

For self-propelled thunniform locomotion, another aspect of kinematics is the detailed characterization of the caudal fin behavior. The simulations show that the cruising velocity increases, as the oscillating amplitude of the caudal fin is raised, and we have obtained the values for *Re* which are inline with the experimental flow regime of typical thunniform swimmers (Dewar and Graham [8] and Wardle *et al*. [52]). Further in the context of the biological reference, the maximum lateral excursion at the tip of the snout computed in this work lies well within the range of 2.3–4.7%*L* reported in Donley and Dickson’s experiment [53]. Based on the current simulations, we also conclude that the dimensionless number characterizing lateral undulation *Sw* of the thunniform fish is dominated by the basic scaling law governing undulatory swimming (Gazzola *et al*. [86]). Moreover, the mean input power decreases as *θ*_*m*_ is raised, along with increasing *V*_*s*_, indicating that the thunniform fish can achieve fast propulsion accompanied by low power requirement through the mode of kinematics. It is interesting to note that the reverse Kármán vortex street in the wake changes its arrangement form such that the transverse flow becomes waker as *θ*_*m*_ is increased. Therefore, the kinetic energy loss in the wake reduces with the rise of *θ*_*m*_ and the efficiency increases, while the Strouhal number decreases gradually and approaches the optimum value of 0.3 reported for efficient oscillatory propulsion of swimming animals (Triantafyllou *et al*. [60, 85]) at *θ*_*m*_ = 35°. In spite of similar area of suction region and vorticity magnitude for various *θ*_*m*_, the swimmer can obtain better propulsive performance with the increased oscillating amplitude that has further tilted the pressure difference between both sides of the fin in the thrust direction.

## Supporting information

S1 Spreadsheet Mesh and time-step sensitivity study for thunniform fish. (XLS)

S2 Spreadsheet Validation of the numerical method. (XLS)

S3 Spreadsheet Temporal variation of forward velocity *V*_*f*_, transverse velocity *u*_*TY*_ and yawing angular velocity Ω_*Z*_. (XLS)

S4 Spreadsheet Time history of total longitudinal force *F*_*l*_. (XLS)

S5 Spreadsheet Fluid forces (moments) of front body, rear body and caudal fin during cruising in one specific cruising period. (XLS)

S6 Spreadsheet Variation in (a) forward velocity and (b) longitudinal force with time for various *A*_*p*_. (XLS)

S7 Spreadsheet Temporal variation of (a) transverse force and (b) yawing moment for various *A*_*p*_. (XLS)

S8 Spreadsheet Variation in output power and efficiency of caudal fin and the whole-fish efficiency with *A*_*p*_. (XLS)

S9 Spreadsheet Time history of (a) forward velocity and (b) longitudinal force for different oscillating amplitudes of the caudal fin. (XLS)

S10 Spreadsheet Variation in (a) transverse force and (b) yawing moment with time for various oscillating amplitudes of the caudal fin. (XLS)

S11 Spreadsheet Variation in output power and efficiency of caudal fin and the whole-fish efficiency with *θ*_*m*_. (XLS)
